# Modeling the development of cortical responses in primate dorsal (“where”) pathway to optic flow using hierarchical neural field models

**DOI:** 10.3389/fnins.2023.1154252

**Published:** 2023-05-22

**Authors:** Anila Gundavarapu, V. Srinivasa Chakravarthy

**Affiliations:** ^1^Computational Neuroscience Lab, Indian Institute of Technology Madras, Chennai, India; ^2^Center for Complex Systems and Dynamics, Indian Institute of Technology Madras, Chennai, India

**Keywords:** optic flow, lateral connectivity, asymmetric Hebbian learning, response similarity matrix, motion processing, multi stage neural network, neural field network

## Abstract

Although there is a plethora of modeling literature dedicated to the object recognition processes of the ventral (“what”) pathway of primate visual systems, modeling studies on the motion-sensitive regions like the Medial superior temporal area (MST) of the dorsal (“where”) pathway are relatively scarce. Neurons in the MST area of the macaque monkey respond selectively to different types of optic flow sequences such as radial and rotational flows. We present three models that are designed to simulate the computation of optic flow performed by the MST neurons. Model-1 and model-2 each composed of three stages: Direction Selective Mosaic Network (DSMN), Cell Plane Network (CPNW) or the Hebbian Network (HBNW), and the Optic flow network (OF). The three stages roughly correspond to V1-MT-MST areas, respectively, in the primate motion pathway. Both these models are trained stage by stage using a biologically plausible variation of Hebbian rule. The simulation results show that, neurons in model-1 and model-2 (that are trained on translational, radial, and rotational sequences) develop responses that could account for MSTd cell properties found neurobiologically. On the other hand, model-3 consists of the Velocity Selective Mosaic Network (VSMN) followed by a convolutional neural network (CNN) which is trained on radial and rotational sequences using a supervised backpropagation algorithm. The quantitative comparison of response similarity matrices (RSMs), made out of convolution layer and last hidden layer responses, show that model-3 neuron responses are consistent with the idea of functional hierarchy in the macaque motion pathway. These results also suggest that the deep learning models could offer a computationally elegant and biologically plausible solution to simulate the development of cortical responses of the primate motion pathway.

## Introduction

Optic flow refers to global motion in the retinal image caused by the motion of the observer relative to the world (Gibson, 1950). It is used to compute useful quantities such as heading direction, which specifies the direction of self-motion relative to the direction of gaze, and the translational and rotational velocity of the observer. The Middle Temporal (MT) area contains many direction-selective cells (Maunsell and van Essen, 1983a,b; Rodman and Albright, 1987) that encode the flow field of the evolving retinal image (Bülthoff et al., 1989; Wang et al., 1989; Newsome et al., 1990; Movshon et al., 1992; Britten et al., 1993; Lappe et al., 1996). In Medial Superior Temporal (MST) area, many neurons respond to spatially extended random dot optic flow patterns (Saito et al., 1986; Tanaka and Saito, 1989; Duffy and Wurtz, 1991b). Cells in MST area have large receptive fields ~15–100° (Duffy and Wurtz, 1991a,b), that respond selectively to expansion, rotation, and combination motion stimuli that are generated due to observer motion (Saito et al., 1986; Tanaka and Saito, 1989; Graziano et al., 1994). MST cells receive their primary input from MT (Desimone and Ungerleider, 1986; Boussaoud et al., 1990) where the initial processing of optic flow involves computation of direction and speed within a small region of the visual field. The emergence of MST and MT cell responses poses an important question: “How can local MT motion estimates be organized into the global selectivity for optic flow that helps in the estimation of heading?” Various models have been proposed to elucidate the possible implementation of optic flow and heading estimation in the area of MST.

### Related modeling studies

Smith et al. (2006) proposed a computational model in which optic flow selectivity is derived by integrating over the MT region where neurons are selective for the local direction of optic flow at each point in the receptive field. However, it does not address how MST cells may facilitate navigation by helping to compute estimates of heading. Lappe and Rauschecker (1993) devised a network model of heading estimation in which a population of neurons codes for a specific heading direction. The line of modeling proposed by Perrone (1992), Perrone and Stone (1994), and Stone and Perrone (1997) took a different view where individual units directly code for heading direction as an early step in the cascade of processing necessary for self-motion perception and navigation. Some authors proposed that there are three main classes of biological models of neural processing at MST: differential motion, decomposition, and template models (Browning et al., 2008). On the other hand, the neural network model for the extraction of optic flow proposed by Tohyama and Fukushima (2005) and Fukushima (2008) suggests a different approach: the vector field hypothesis that any flow field can be mathematically decomposed into elementary flow components such as divergence and curl.

Most of these findings provide compelling evidence that heading perception is based on the evolution of the optic flow field but are not especially informative about the nature of the underlying neural mechanisms such as temporal dynamics that arise due to the integration of information over time. This motivated us to identify the role of temporal dynamics in visual information processing. Consequently, we proposed a neural field network to simulate the direction selectivity of V1 cells and pattern selectivity of MT cells in our earlier published work (Gundavarapu et al., 2019). In this study, we extended our earlier neural field model into direction selective mosaic network (DSMN) and velocity-selective mosaic network (VSMN), which can process optic flow sequences. We framed our primary objective as designing a biologically plausible model that can simulate the optic flow selective responses of MSTd neurons and we came up with model-1 and model-2, each can successfully recognize the type of flow present in the given sequence. Later, we extended our objective toward the development of a more generalized model of motion processing with the inspiration of the following studies.

### Recent deep learning approaches to modeling neuron selectivity in the visual system

Biological realism is the primary concern of most of the neuroscience models (Hubel and Wiesel, 1962; Simoncelli and Heeger, 1998; Rousselet et al., 2002; Rust et al., 2006; Serre et al., 2007; Pack and Born, 2008). These models are designed to account for anatomical and neurophysiological data and did not scale up to solving real-world tasks. Currently, feed-forward convolutional neural networks (CNNs; LeCun et al., 2015), are the state-of-the-art for object classification tasks such as ImageNet, on occasion surpassing human performance (He et al., 2015). Recently several studies (Agrawal et al., 2014; Güçlü and van Gerven, 2015; Kriegeskorte, 2015) have begun to assess the convolutional neural network as a model for biological vision systems by comparing the internal representations and performance levels between artificial and biologically realistic neural network models. Studies also showed (Kriegeskorte, 2015) that deep convolutional neural networks trained on object recognition tasks not only have architectural similarities but also learn representations that are similar to the representations of the neurons in the ventral pathway. However, their suitability and performance on the dorsal-stream regions is an open area of research, which lead us to develop a generalized model of a motion processing system.

**Figure 1:**
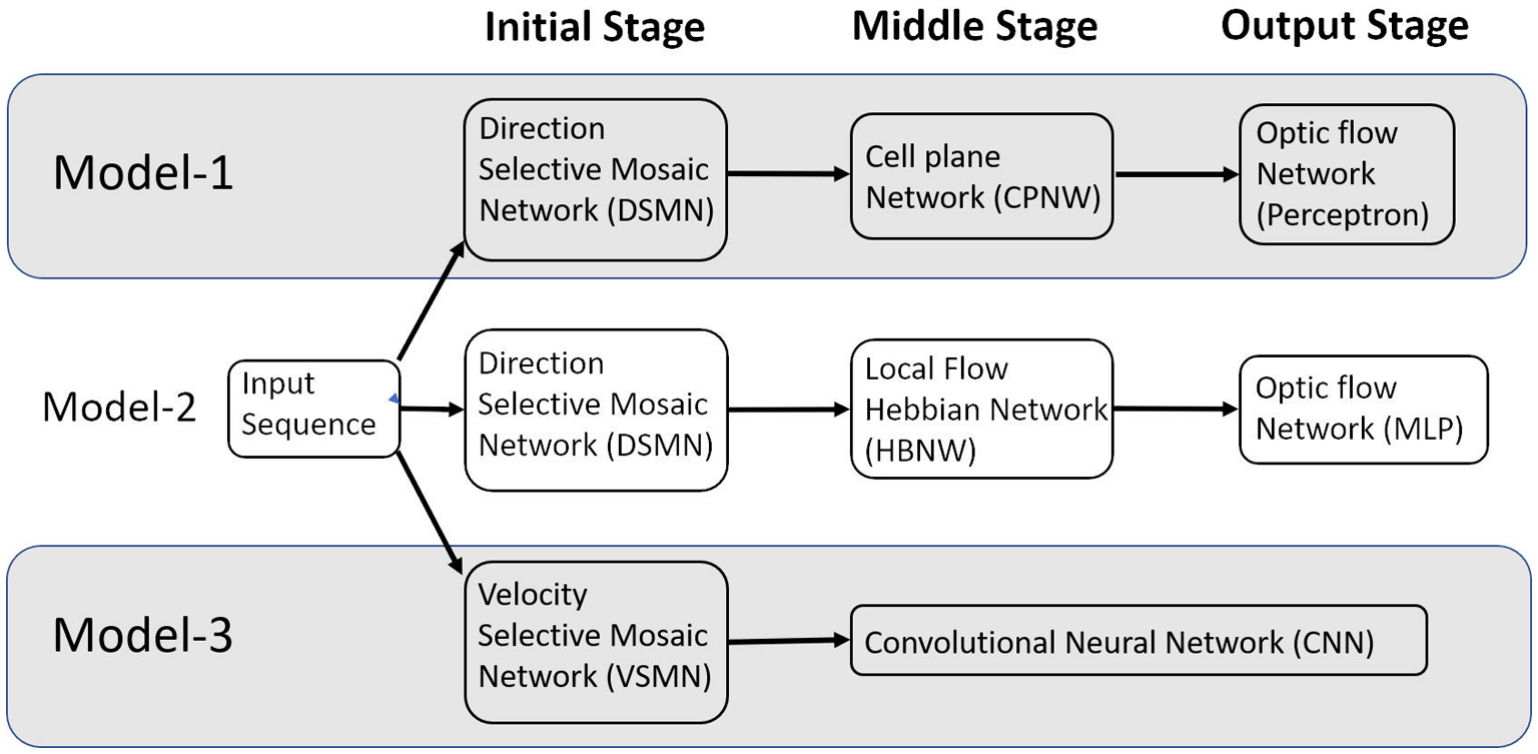
The multi-stage neural network models composed of the initial, middle, and output stages that are simulating the neurons at V1, MT, and MSTd, respectively.

In this paper, we describe (i) a competitive learning algorithm/design (model-2) that shows the emergence of optic flow sensitivity and (ii) a deep neural architecture (model-3) that can learn motion-related properties similar to the representations of neurons in MT/MST. Section 2 “Model architecture and learning rules” describes the architecture of the three models. Even though we described it as three distinct models, they have some common components; components that differ between the models perform equivalent functions (Figure 1). The main structure of these models is a multi-stage neural network composed of the initial stage simulating the direction-selective neurons of V1, the middle stage simulating translational motion selective neurons of MT, and the output stage simulating the optic flow selective MST neurons. An important common feature of all these proposed models is the presence of 2D layers of neurons with lateral connections trained by asymmetric Hebbian learning (stage-1). The combination of lateral connectivity and asymmetric Hebbian learning provides an opportunity for extracting motion information. Section “Model architecture and learning rules” also discusses the training procedure of the three network models. Section “Results” shows the simulation results and compares the performances of the model with the biological properties of the MST neurons. Section “Discussion” and Section “Conclusion” consist of a discussion and conclusion, respectively.

## Model architecture and learning rules

This section first describes various components/sub-networks used in all three models and then presents the complete pipeline for all three models. Before that, we list out various physiological evidence used in designing these models.

### Physiological pieces of evidence used in designing the model

Neurophysiological studies (Saito et al., 1986; Tanaka and Saito, 1989; Graziano, 1990; Duffy and Wurtz, 1991a,b) have found that most of the neurons in the dorsal part of the medial superior temporal (MSTd) area of the visual cortex in the primates’ brain are responsive to different types of optic flow stimuli. It also has been found that MST receives strong projections from the middle temporal (MT) area (Maunsell and van Essen, 1983a,b; Ungerleider and Desimone, 1986) where the neurons selectively respond to the orientation and velocity of the visual stimuli (Albright, 1984; Rodman and Albright, 1987). It is therefore natural to assume the MT area to be the preprocessing stage to the optic flow processing taking place in MST area. There is physiological evidence that translational motion is computed in area MT (Movshon et al., 1985) while radial and rotational motions are first seen in the response properties of cells in MSTd (Tanaka et al., 1989; Tanaka and Saito, 1989; Duffy and Wurtz, 1991a,b). Thus, according to this view, optic flow stimuli are processed serially, starting in the striate cortex with the analysis of motion in local parts of the visual field by direction-selective cells with small receptive fields.^1^ This local motion information is globally integrated into area MT by cells with larger receptive fields, which compute pattern motion—in this case translational motion. Finally, global radial and rotational motion is encoded by MSTd cells with much larger receptive fields based on their MT input. The selective responses of some MSTd cells are said to be position dependent, while those of others are position independent. In support of this view, it was suggested by several researchers (Saito et al., 1986; Tanaka and Saito, 1989) that the receptive field of an MST cell responsive to circular or radial motions is composed of a set of directionally selective MT cells arranged following the pattern of that optic flow component. Thus, the input MT cells would be arranged radially in the case of an expansion/contraction MST cell, or arranged circularly in the case of a rotation MST cell. Keeping these earlier proposals in view, to understand the responses of MST neurons and to explain how motion information is extracted to discriminate the type of optic flow, we proposed an architecture composed of three stages: the initial stage consists of direction-selective neurons that are trained to respond to the direction of motion of dots present in a given receptive field; the middle stage neurons are trained with translational sequences so that each neuron is selective to the direction of motion of local translational motion; neurons in the output stage are tuned to the type of the optic flow present in the input sequence. Compared to the algorithms of optic flow analysis proposed by the computer vision community, the proposed modeling approach is more physiologically plausible and can account for some of the response properties of MSTd neurons. Figure 1 shows the schematic representation of the three models proposed.

### Direction selective mosaic network

**Figure 2:**
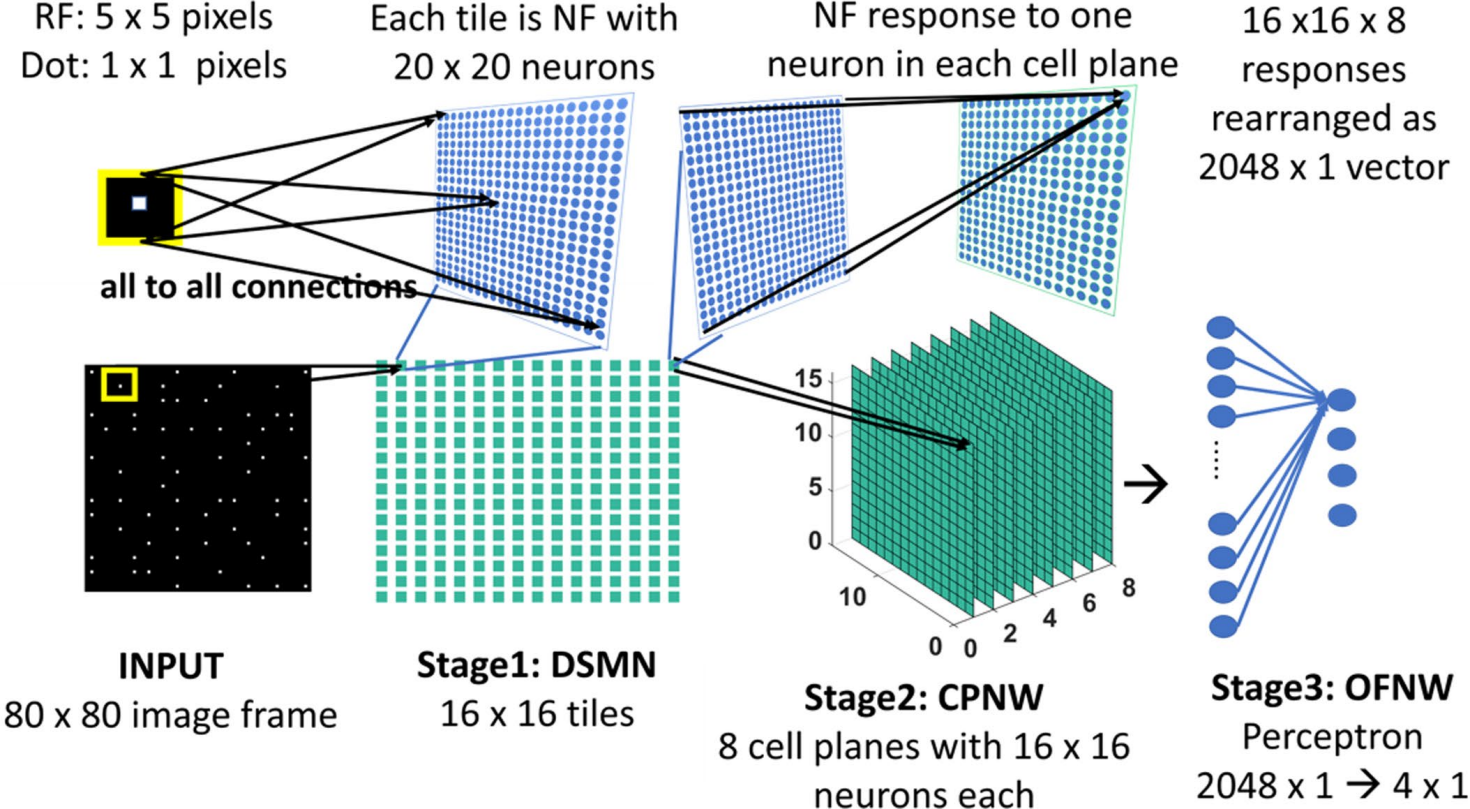
Model-1 architecture. All the neurons in NF receive the same input of size 5 × 5 but maintain random initial afferent weights. Response of one NF is passed to one cell-plane neuron. Thus, each cell-plane neuron maintains 400 (20 × 20) afferent connections with its input which were adopted during training. The response on eight cells-planes is converted into 1D vector and then concatenated to pass to OFNW/perceptron. OFNW is trained to classify four types of flow sequences.

The initial stage consists of a 16 × 16 mosaic of 2D arrays (“tiles”) of neurons, named Direction Selective Mosaic Network (DSMN). Every tile is an independent neural field (NF) wherein the neurons respond preferentially to the direction of motion of a dot present within their receptive fields. The neurons in an NF have lateral connections, thereby making the response of the NF neurons dependent on history. The input to DSMN can be visualized as 16 × 16 non-overlapping image patches of size 5 × 5. Thus, the size of the input image is 80 × 80 [= (16 × 16) (5 × 5)]. Each NF is composed of 20 × 20 neurons, receiving common input from a single 5 × 5 patch of the input image (Stage1: DSMN in Figure 2). Although all the neurons in a given NF respond to the same 5 × 5 patch of their input image, they develop distinct selectivities since they have random initial weights, and the initial differences are amplified by the competitive dynamics within the NF, as explained in more detail in the following section. As a result of training, NF neurons were clustered into various populations/ groups in such a way that each is selective to a specific direction of motion of a dot.

#### Training procedure: the neural field

As shown above, the input image provided to DSMN is of size 80 × 80 pixels. Each NF is composed of 20 × 20 neurons. The neurons in a given NF receive common input from a 5 × 5 window of an input image. The initial response of NF neuron (*i, j*) is calculated as,


(1) $$A_{ij}=\sigma \left(\gamma_{aff}*\sum_{rs}\left(W_{ij,rs}\;X_{rs}\;\right)\;\right)$$

where *X* is a 5 × 5 image window of neuron (*i, j*). *W* is the afferent weight matrix of neuron (*i, j*). Let *X_rs_* be the pixel position in the image window then *W_ij, rs_* is the afferent weight connection from (*r, s*) to (*i, j*). *γ_aff_* is a constant scaling factor and is initialized before training begins. *σ* is a piecewise linear sigmoid activation function.

The response of an NF neuron is influenced by both afferent inputs and the inputs from the lateral connections. Therefore, though the initial response is dominated by the afferent input, *A_ij_*, subsequently the response is further modified by the lateral connectivity of NF neurons. Lateral interactions are characterized by ON-center, OFF-surround neighborhoods. Two types of lateral connections exist (i) Excitatory laterals that connect neuron (*i, j*) with neuron (*k, l*) within a given neighborhood. The excitatory neighborhood is specified by the radius parameter *r_exc_* and is initialized before training begins. The *r_exc_* value is uniform for all the neurons within and across NFs. (ii) Inhibitory laterals inhibit the response of the neuron (*i, j*). It should be noted that the neuron (*i, j*) maintains inhibitory connections only with the neurons that are present outside the radius *r_exc_* and inside the radius *r_inhb_*.

For several time steps *“s”* (settling time), the response of the neuron (*i, j*) is modified by afferent and lateral interactions that take place simultaneously.


(2) $$\begin{array}{l} \eta_{ij}\left(s\right)=\sigma (A_{ij}+\gamma_{exc}\sum_{kl}\eta_{kl}\left(s-1\right)*E_{ij,kl}-\gamma_{inhb}\\ \sum_{kl}\eta_{kl}(s-1)*I_{ij,kl}) \end{array}$$

where *η_ij_* stands for the activity of the neuron (*i, j*), *E_ij,kl_* and *I_ij,kl_* are excitatory and inhibitory weights from the neuron (*k, l*) to (*i, j*) that are randomly initialized before training begins. *A_ij_*, as defined in Equation 1, is the total afferent input into the neuron (*i, j*). The relative strengths of excitatory and inhibitory lateral effects are controlled by the constant scaling factors *γ_exc_* and *γ_inhb_*.

At the end of “*s*” time steps, NF response settles down and all three types of weights (afferent, excitatory laterals, and inhibitory laterals) are updated. Let “*t*” represent the time of presentation of the current frame to NF with the assumption that at *t* = 1, frame-1 is presented. The afferent weight connections are adapted using the symmetric Hebbian rule (Equation 3) and the lateral weight connections are adapted using the asymmetric Hebbian rule (Equation 4).


(3) $$\Delta W_{ij,rs}\left(t\right)=\alpha_{aff}*X_{rs}\left(t\right)*\eta_{ij}\left(t\right)$$

where $\alpha_{aff}$ is the learning parameter for afferent weight connection. *X* is a 5 × 5 image window from which neuron (*i, j*) receives input. *W_ij, rs_* is the afferent weight between the pixel position (*r, s*) and the neuron (*i, j*). *η_ij_*(*t*) is the activity of neuron (*i, j*) after the settling process for the current frame “*t*.” The weights are updated after the presentation of each image in the input sequence as follows:


(4) $$\Delta W_{ij,kl}\left(t\right)=\alpha_{lat}*\max\left(0,\left(\eta_{ij}\left(t\right)-\eta_{ij}\left(t-1\right)\right)\right)*\eta_{kl}\left(t-1\right)$$

(5) $$W_{ij,kl}^{new}\left(t\right)=\frac{W_{ij,kl}^{old}\left(t\right)+\Delta W_{ij,kl}\left(t\right)}{\sum_{u}\left(W_{ij,kl}^{old}\left(t\right)+\Delta W_{ij,kl}\left(t\right)\right)}$$

where *ɳ_ij_*(*t*) is the settled activity on the neuron (*i, j*) produced in response to the current frame (the frame presented at time “*t*”), *ɳ_ij_*(*t-1*) is the settled activity on the neuron (*i, j*) for the previous frame. *α* is the learning rate. Separate learning parameters (in place of *α_lat_*) were used for excitatory ($\alpha_{exc}$) and inhibitory ($\alpha_{inhb}$) connections. All three types of weight connections are normalized separately as shown in Equation 5 to prevent the neural activity from growing out of bounds. Various parameters used in the simulation are specified in Table 1.

**Table 1:** Parameters used to train tiles (NF) in DSMN and VSMN.

Parameter	Tile in DSMN	Tile in VSMN
NF Dimension	20 × 20	48 × 48
Receptive field	5 × 5	8 × 8
r_exc_	2	2
r_inhb_	5	4
γ_aff_	1	1
γ_exc_	21.6	50
γ_inhb_	1	1.5
α_aff_	0.05	0.05
α_exc_	0.05	0.05
α_inhb_	0.05	0.05
Time step(s)	10	10
Epochs	500	250
Image size	80 × 80	80 × 80

Note that the training procedure described above is for one tile (NF) that takes input from a 5 × 5 window of an input image. 16 × 16 array of such tiles was trained sequentially one after the other by presenting an input image sequence consisting of 15 frames, each of size 80 × 80.

### Cell plane network

The cell plane, according to Tohyama and Fukushima (2005), is a group of cells in which all cells have receptive fields of identical characteristics, but the locations of the receptive fields differ from cell to cell. For example, a group of cells with the same preferred moving direction is referred to as a cell-plane. The proposed CPNW consists of 8 cell planes (Stage 2: CPNW in Figure 2), each responding preferentially to the translational motion of a particular direction. This translational motion selectivity of each cell-plane is achieved through training. For simplicity, and also for the fact that MT neurons are not sharply tuned for speed, only the direction of each flow field (DSMN response) was used to represent motion in the MT stage.

#### Training procedure

Each cell-plane consists of a 2D array of neurons of size 16 × 16; there are *N*_cp_ (=8) cell-planes used in model-1. Neuron (*p, q*) in the *n^th^* cell-plane receives afferent input from all the neurons of (*p, q*)th tile. Note that DSMN has 16 × 16 tiles, where each tile is made up of 20 × 20 neurons. The activity of the neuron (*p, q*) in the *nth* cell-plane is computed using Equation 6 and subsequently its afferent weights are updated using Equation 7. All the initial afferent weights are set randomly. The eight cell-planes were trained sequentially one after the other by considering different stimuli sets. In other words, cell-plane-1 is trained using stimuli set consisting of dots translated coherently in 0°, cell-plane-2 is trained using dots translated coherently in 45°, and so on. Thus, each cell-plane is trained independently using dots moving coherently in eight different directions. At the end of the training, the eight cells-planes develop selectivities to eight directions of dot motion.


(6) $$C_{pq}^{n}=\sigma \left(\overset{20}{\underset{r=1,s=1}{\sum }}W_{pq,rs}^{n}Z_{rs}\right)$$

where *σ* is a sigmoid function. C^n^_pq_ is the response on the neuron (*p, q*) in the *n*th cell plane. Z (20 × 20) is the (*p, q*)th tile response in DSMN. W^n^_pq, rs_ represents the afferent connection from the neuron (*r, s*) within (*p, q*)th tile to the (*p, q*)th neuron in the *n*th cell-plane.


(7) $$W_{pq,rs}^{n}\left(\mathrm{new}\right)=\frac{W_{pq,\mathrm{rs}}^{n}\;\left(\mathrm{old}\right)+\alpha_{aff}*Z_{rs}*C_{pq}^{n}}{\sum_{rs}\left(W_{pq,\mathrm{rs}}^{n}\;\left(\mathrm{old}\right)+\alpha_{aff}*Z_{rs}*C_{pq}^{n}\right)}$$

where *α_aff_* is the learning parameter, set to 0.05 during the simulation. Weights are updated for each sequence presentation. Updated weights are normalized to prevent them from going out of bounds.

### Hebbian network

**Figure 3:**
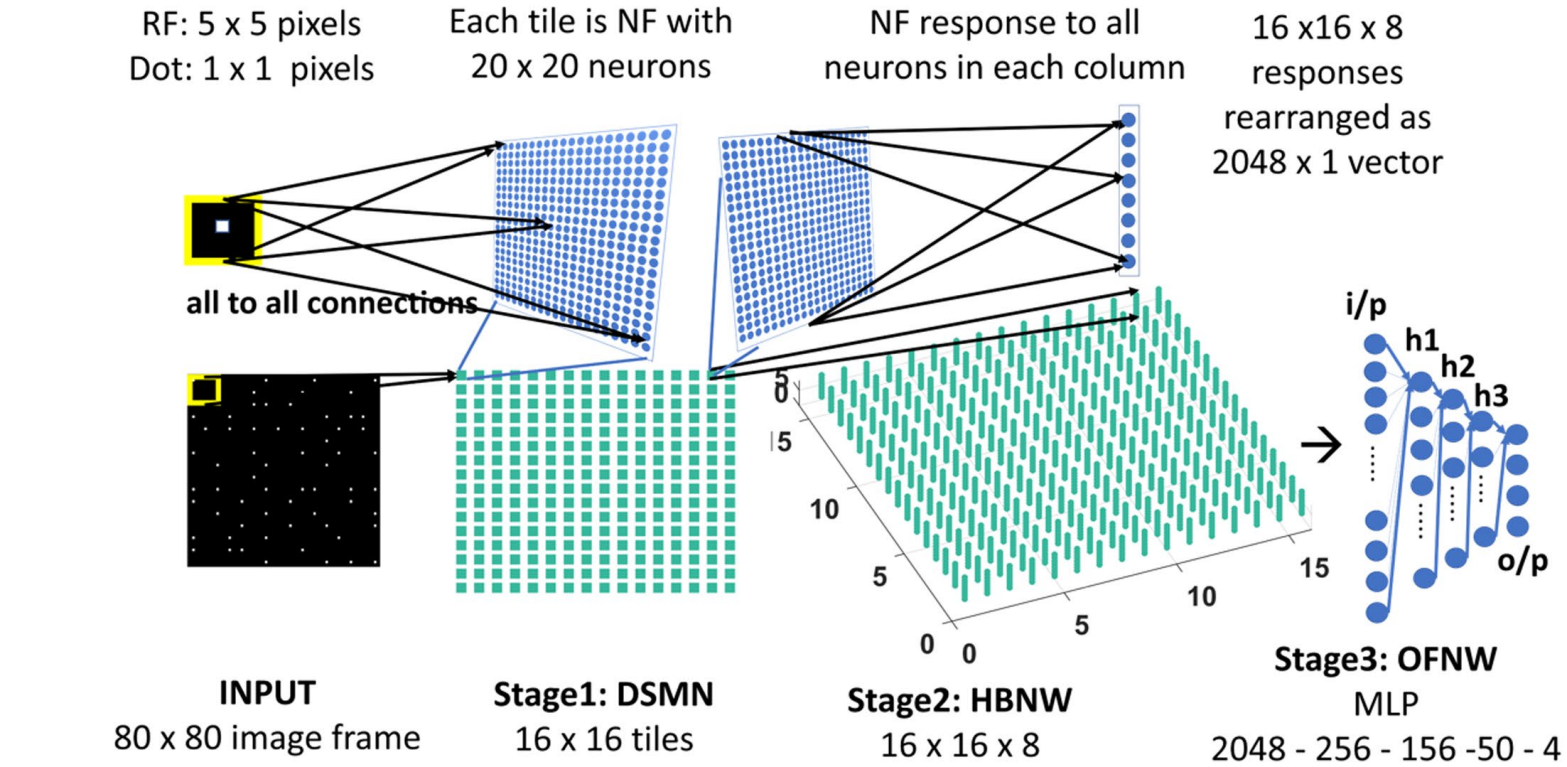
Model-2 architecture. DSMN (consisting of 16 × 16 tiles) is similar to the one described in model-1. 16 × 16 NF responses were feed forwarded to 16 × 16 columns in HBNW. HBNW response is rearranged as 2,048 × 1 vector before it is passed to the output stage-multi-layer perceptron (MLP) where the neurons are trained to recognize the type of optic flow present in the input sequence.

The neurons in the Hebbian Network (HBNW) are arranged similarly to CPNW neurons, but trained differently using competitive learning (Fukushima and Miyake, 1982; Fukushima, 1988; Fukushima et al., 1997). In this learning, mode neurons compete in a winner-takes-all fashion, and the neuron receiving the largest input wins the competition and the winner gets to modify its weights. Thus, neurons learn to respond to the inputs whose preferred direction best fits the local motion direction in the input. HBNW is composed of 16 × 16 × 8 neurons (Stage2: HBNW in Figure 3) and is regarded as a 16 × 16 array of columns of neurons with 8 neurons in each column. A given column of neurons at location (*m, n*) receives a shared input *Z*_rs_ from a tile (*m, n*) in DSMN (that consists of 16 × 16 tiles). Similar to Equation 6, the response of a neuron (*m, n, k*) is computed as a scalar product of the response of the tile (*m, n*) and the afferent weight matrix of the neuron (*m, n, k*; eqn. Shown again below the paragraph). All afferent weights are initialized randomly, accordingly the neurons across *the* (*m, n*) column respond differently. The neuron in column (*m, n*) whose afferent weight matrix is closest to the input [which is the response of the tile (*m, n*) in DSMN] will produce the highest activity, subsequently becomes a winner and its weights get updated following Equation 8.


$$C_{mn}^{k}=\sigma \left(\overset{20}{\underset{r=1,s=1}{\sum }}W_{mn,rs}^{k}Z_{rs}\right)$$

$$\delta_{m,n,k}=\left\{\begin{array}{c} 1\;\;ifneuron\;\left(m,n,k\right)\;iswinner\\ 0\;\;otherwise\; \end{array}\right.$$

(8) $$W_{mn,rs}^{k}\left(\mathrm{new}\right)=W_{mn,\mathrm{rs}}^{k}\;\left(\mathrm{old}\right)+\alpha_{aff}*Z_{rs}*C_{mn}^{k}*\delta_{m,n,k}$$

where *σ* is a sigmoid function. During weight adaptation, the afferent weights of the winner alone were normalized. C^k^_mn_ is the response of the neuron (*m, n, k*). Z (20 × 20) is the (*m, n*)th tile response in DSMN. Note that (*m, n*)th tile is NF made up of 20 × 20 neurons. *Z_rs_* refers to the response on (*r, s*) neuron within NF in other wards the tile (*m, n*) in DSMN. W^k^_mn, rs_ represents the afferent connectivity from the neuron (*r, s*) within (*m, n*)th tile to the (*m, n, k*)th neuron in HBNW. *α_aff_* is a learning parameter for afferent weights, whose value is set to 0.05 during the simulation.

#### Difference between HBNW and CPNW

The arrangement of the receptive fields of the neurons in both networks is the same. CPNW neurons are grouped into 2D arrays and neurons in each group learn to respond preferentially to the same translational motion direction. Such grouping does not exist in HBNW, instead, neurons in each column compete in a winner-take-all fashion. During training, different CPNW 2D arrays have access to different stimulus sets. On the other hand, every neuron in HBNW has access to a continuum of input stimuli. Due to competitive learning, each neuron learns to discover a unique salient feature (direction of motion) present in the small part of the input. At the end of the training, a continuum of input stimuli is divided into a set of distinct clusters, where each cluster is represented by a particular set of HBNW neurons.

### Optic flow network

A well-known perceptron and multi-layer perceptron with three hidden layers were used to implement the Optic Flow Neural Network (OFNW) in model-1 and model-2, respectively. Both networks are developed and trained in MATLAB 2015.

#### Multi-class perceptron

The multi-class perceptron implemented as OFNW has an input layer followed by an output layer. The response of the CPNW is rearranged as a 1D vector before it is fed into the OFNW. The perceptron output layer consists of four nodes, each being trained to recognize the type of optic flow (expansion, contraction, clockwise rotation, and anti-clockwise rotation) present in the given input sequence.

Let the training examples be (*X_1_, y_1_*), (*X_2_, y_2_*),…,(*X_n_,y_n_*), where *X_i_* is an input vector and the labels *y_i_* are drawn from the set *{l_1_, l_2_,... l_k_}*. Let the set of weight vectors to be learned are *{W^1^, W^2^,...,W^k^}*, then multiclass perceptron can be implemented as


$$D=argmax_{y\in \left\{1,\ldots ,k\right\}}\;\left(W^{y}.x\right)$$

$$if\;D\neq y_{i}$$

$$\;W^{r}\leftarrow W^{r}+\tau_{r}x_{i}$$

where,


(9) $$\tau_{r}=\left\{\begin{array}{c} +1\;\;if\;r=y_{i}\\ -1\;\;if\;r=D\\ 0\;\;otherwise \end{array}\right.$$

#### Multi-layer perceptron

Multi-layer perceptron network consists of three hidden layers, and is trained using a regular backpropagation algorithm which was described in various studies (Rumelhart et al., 1986; Hornik et al., 1989; LeCun et al., 1992; Bishop, 1995).

### Velocity selective mosaic network

**Figure 4:**
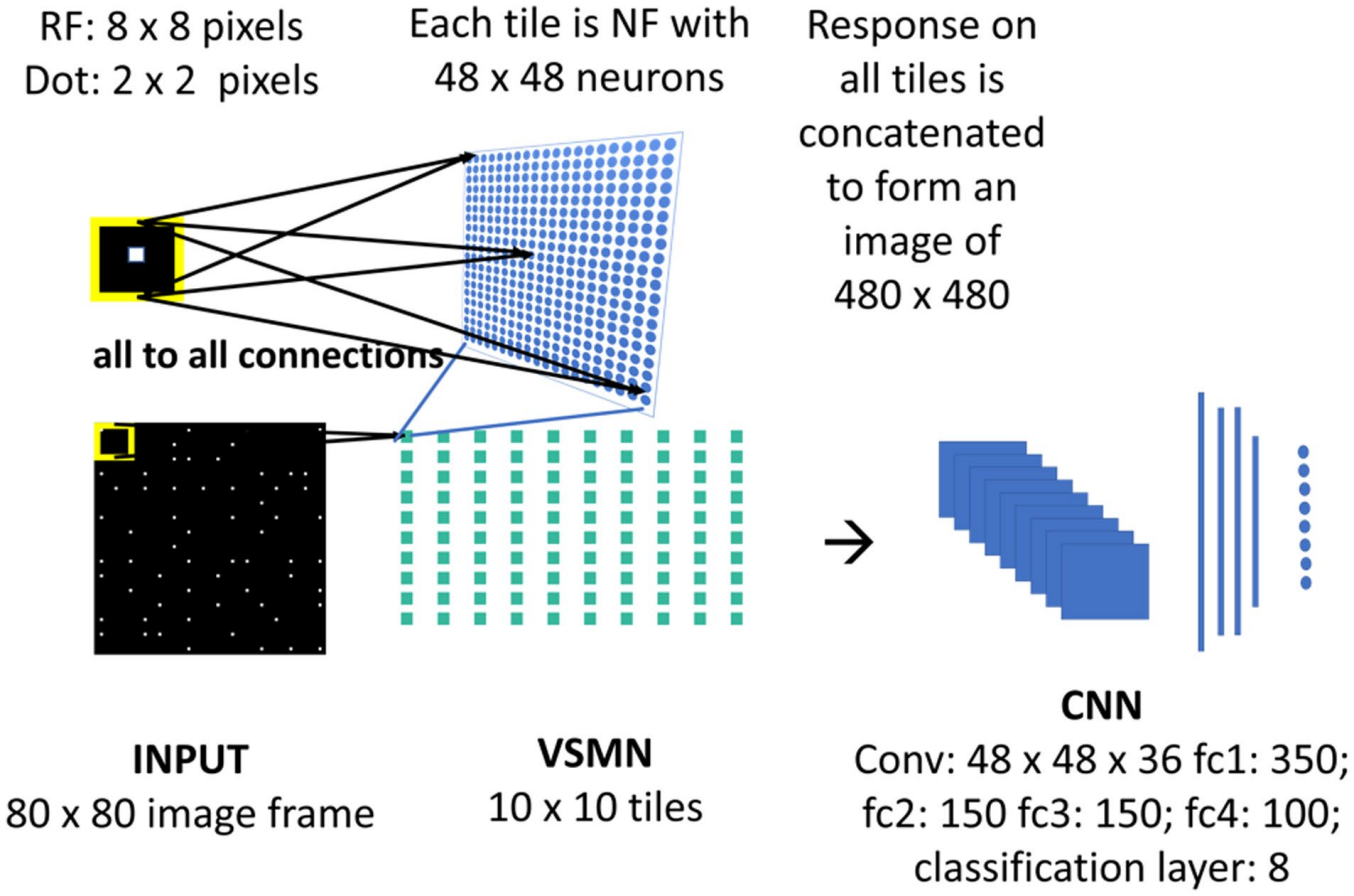
Model-3 architecture. The initial stage consists of VSMN that takes the input from image sequence. Each tile in VSMN is NF (48 × 48) where neurons are trained to encode direction and speed of a dot (2 × 2) within RF (8 × 8). The VSMN response obtained at the end of presentation of sequence is used to train CNN after concatenating the 10 × 10 tiles responses as a 2D image.

The velocity Selective Mosaic Network (VSMN) used in model-3 (Figure 4) is nearly the same as model-1 except it is formed out of 10×10 tiles and each tile is of size 48 × 48. Equation 1 and Equations 3–5 are used for the calculation of VSMN initial response and weight adaptation. The equation for the calculation of settled response, Equation 2, is modified. The input sequences generated to train the DSMN have fixed speeds. Equation 10 is a modified form of Equation 2 to make the network recognize variable speeds along with the direction of motion, i.e., to recognize velocity. In our simulations, we tried various scaling values for δ (see Equation 12) ranging from 0.1 to 0.001. At higher values δ, the network fails to distinguish speed. At lower values of δ, the network response is unstable during the presentation of the sequence. In other words, the lateral interactions could not produce unique activity patterns in NF to encode the speed feature.


(10) $$\begin{array}{l} \eta_{ij}\left(s\right)=\sigma (A_{ij}+\gamma_{exc}\underset{kl}{\sum }\eta_{kl}\left(s-1\right)*\\ E_{ij,kl}-\gamma_{inhb}\underset{kl}{\sum }\eta_{kl}(s-1)*I_{ij,kl}-\delta *\eta_{ij}(s-1)) \end{array}$$

Unlike in DSMN where each tile is trained separately, in VSMN, each tile or NF is trained using input sequences (frame size 8 × 8) made up of 2 × 2 tiny squares moving in eight directions and in each direction at two speeds. Thus, the training set of VSMN contains 16 sequences. The weights of a trained tile are copied to the remaining tiles in VSMN, to overcome the computational overhead.

### Convolutional neural network

Velocity Selective Mosaic Network response generated at the end of the presentation of the entire sequence is used as input to the CNN. The responses on 10 × 10 tiles are concatenated to form an image of size 480 × 480. CNN is made up of one convolutional layer (with 36 feature maps) followed by four fully connected layers and a classification layer. CNN is trained to recognize the type of optic flow along with its speed (8 classes = 4 flow types × 2 speeds). The design and simulation of the deep network are carried out using MATLAB 2020a deep learning toolbox.

### Pipeline of all the three models

#### Model-1

As shown in Figure 2, the three stages (DSMN, CPNW, and perceptron) in model-1 are trained one after the other, in three steps, using the procedure described in the previous sections. In the first step, each NF in DSMN is trained using the dot (1 × 1) moving in eight directions such that the NF neurons develop direction-selective responses. In the second step, CPNW is trained with translational sequences moving in eight directions (0, 45, 90, 135, 180, 225, 270, and 315°) such that neurons in each cell-plane respond maximally to specific translational motion direction. Note that while training CPNW, the DSMN weights are fixed and only its responses are forwarded. In the third step, the perceptron is trained to recognize the type of optic flow by using four types (zoom in, zoom out, clockwise, and anti-clockwise) of optic flow sequences, while keeping DSMN and CPNW weights fixed.

#### Model-2

As shown in Figure 3, model-2 consists of three stages—DSMN, HBNW, and MLP and each are trained with moving dot sequences, translational sequences, and optic flow sequences, respectively, in three steps similar to model-1.

#### Model-3

As shown in Figure 4, model-3 consists of two subnetworks—VSMN and CNN. VSMN is trained using the dot (2 × 2) moving in eight directions such that the NF neurons develop direction sensitivity along with speed-selective responses (i.e., velocity). These VSMN weights are fixed and only its responses are used while training the CNN. CNN is trained to recognize the type of flow (eight classes: 4 flow types × 2 speeds) present in the given input sequences. The training set of CNN is made up of dots that are allowed to make rotational (clockwise and anti-clockwise) and radial (inward and outward) trajectories.

For a clear understanding of all the three models, the details about the preparation of training and test sets and the network performance were provided along with the results in Section 3.

### Correlation measures used to construct response similarity matrix

Pearson correlation measures (Kpolovie, 2011; Emerson, 2015) and Euclidean distance measures (Dokmanic et al., 2015) were used to construct response similarity matrices. The Pearson correlation coefficient *r_xy_* is a statistical measure of the degree of linear correlation between the two variables *x* and *y*. *r_xy_* takes values in the closed interval [−1, +1] (Kpolovie, 2011; Emerson, 2015). The value *r_xy_* = +1 represents a perfect positive correlation between x and y, *r_xy_* = −1 represents a perfect negative correlation between x and y, whereas the value *r_xy_* = 0 indicates that no correlation.

### Creating input stimuli

#### Spatial distribution

For model-1 and model-2, moving dot sequences were created by positioning 64 white dots on a black background of size 80 × 80 pixels with a density constraint that each 10 × 10 window typically accommodates only one dot. Each sequence is comprised of 15 frames. For model-3, dot stimuli were created by positioning 100 tiny white squares of size 2 × 2 pixels upon a black square grid of size 80 × 80 pixels with a constraint that each 8 × 8 window can accommodate only one tiny square at any given time. Each sequence is comprised of 10 frames.

#### Translational motion

Each dot configuration is moved (displacing *x*, *y* coordinates) in eight directions (θ): 0, 45, 90, 135, 180, 225, 270, and 315°. The translational motion is incorporated in 15 or 10 frames as specified above, and each dot configuration adds eight translational trajectories to the training set. If the dot exceeds the square boundary of the frame, it is wrapped around to reappear on the opposite side of the frame; thus, the dot density across the frames was kept constant. The horizontal and vertical displacement of a dot to incorporate translational motion is calculated by the Equation 11.


$$x_{i}\left(t+1\right)=x_{i}\left(t\right)+v\;\;\cos\left(\alpha \right)$$

(11) $$y_{i}\left(t+1\right)=y_{i}\left(t\right)+v\;\sin\left(\alpha \right)$$

where α represents the direction of motion and the local speed is defined by “*v*.” In the case of input stimuli for model-1 and 2, *v* takes only a single value (=1) and for model-3 *v* takes two values (=1,2).

#### Optic flow motion

Each dot configuration is allowed to move along circular (clockwise, anti-clockwise) and radial (expansion, contraction) trajectories to create different flow sequences. Thus, each dot configuration adds four flow patterns to the training set. Let m and φ be the magnitude and orientation components of a dot at (*x, y*). Then, the trajectory of radial and circular motion is defined using the Equations 12, 13. Note that for radial trajectory magnitude (m) varies and for circular motion orientation (φ) varies.


(12) m(t+1)=m(t)+vcosθ

(13) φ(t+1)=φ(t)+vsinθ

where *v* defines the local speed; *θ* defines the direction of flow, and takes the values 0 for expansion, π for contraction, −π/2 for clockwise rotation, and π/2 for anti-clockwise rotation. Here also for models 1 and 2, *v* takes only a single value (= 1) and for model 3, *v* takes two values (=1,2).

## Results

### Model-1

#### DSMN response to translational dot sequences

**Figure 5:**
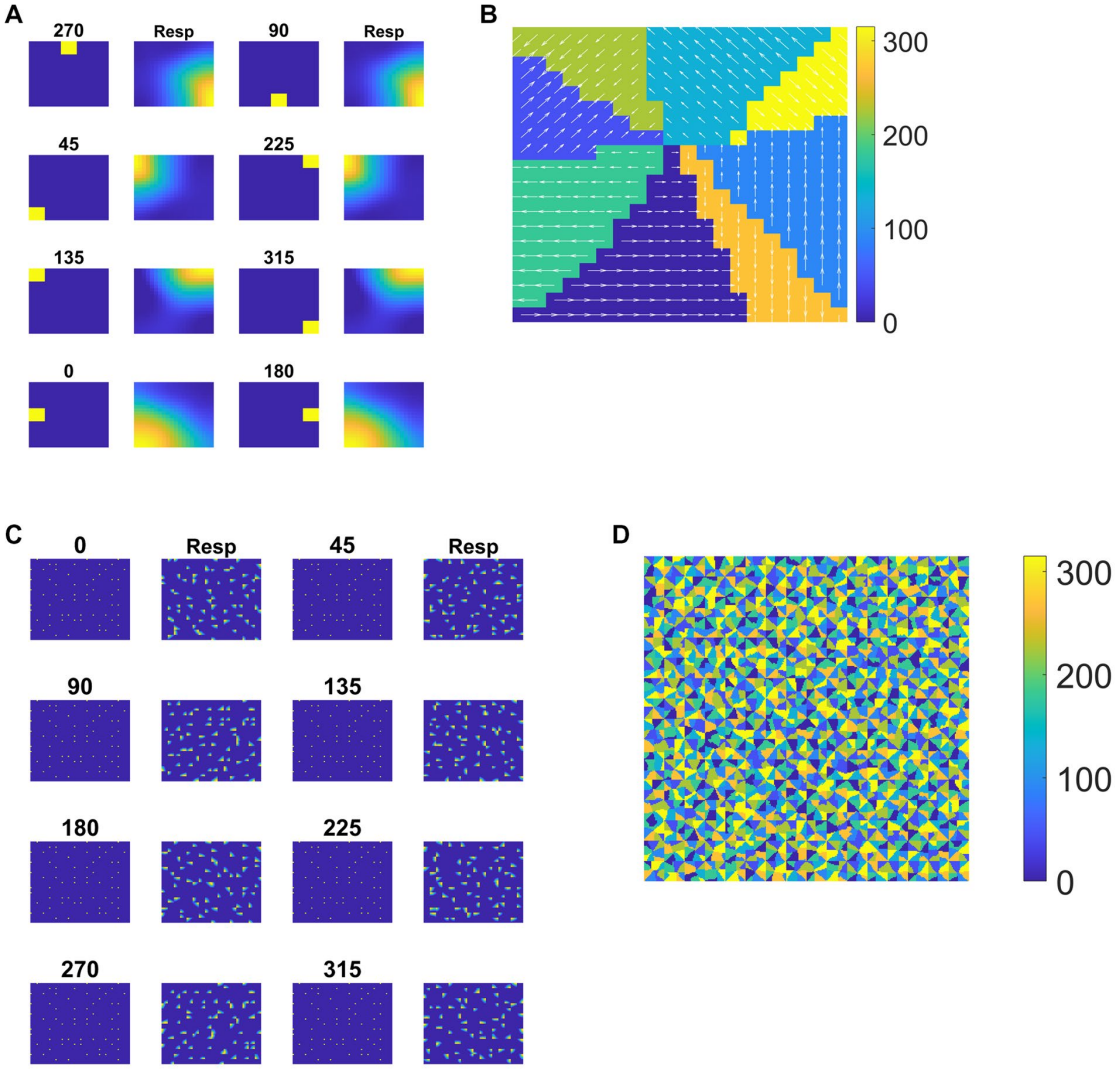
Neural field network and DSMN response. First and third columns in **(A)** display the first frame (5 × 5) of an input sequence and second and third columns display the corresponding NF (20 × 20) activity. **(B)** shows the direction selective map for one tile. In **(C)**, first and third columns show the frame (80 × 80) of a translational sequence and the response it elicits in DSMN (16 × 16 tiles) is plotted in second and fourth columns. “Resp” column shows the concatenated response in all the tiles of DSMN. **(D)** Displays DSMN’s direction selectivity map (concatenated direction selective maps of all 16 × 6 tiles). Each color on the map shows the neuron population with direction preference as specified in the color bar.

Each tile in the DSMN is an NF. Each NF is trained with a dot (1 × 1) moving in eight directions (0, 45, 90, 135, 180, 225, 270, and 315°) from three different initial positions. Thus, the training set is made up of 24 (8 directions × 3 positions) sequences. Trained NF response to the dot moving in eight directions and the corresponding direction selective map is shown in Figures 5A,B. Even though all the tiles are trained to encode the direction of motion of a dot present within their receptive field, the neuron preferences across the tiles vary due to random initial afferent and lateral connections. That is neurons at a specified location (*i, j*) in all the tiles, do not always respond in the same direction. Various network and learning parameters used in the simulation are given in Table 1. Figure 5D represents the map of direction selectivity of DSMN. By comparing the color patches in the maps produced by each tile, one can understand that different neuronal populations are active in different tiles in response to the translational dot pattern moving in a specific direction. Each colored patch indicates the direction preference of the neuronal population. Figure 5C represents the response of DSMN to the translational sequence moving in 8 different directions. The neural responses produced by different tiles in DSMN are concatenated and displayed in the “Resp” column in Figure 5C.

#### Two-stage network response/CPNW response to the translated motion

Here we present simulated results of the CPNW, which is the second stage of model-1. As described earlier, the cell-plane network (CPNW) takes responses from DSMN and is trained by repeatedly presenting translational motion sequences to DSMN (see sections 2.3 and 2.8). Since the learning takes place in the different cell planes independently (neurons in different cell planes do not compete with each other), the responses of the cell planes are similar but not identical. Here we used eight cell planes to encode eight different motion directions. We could also choose to use more cell planes to model a variety of MT and MST cells. Fifteen different initial dot configurations were translated in eight directions to make 120 sequences, which were divided into a training set (10 × 8 = 80) and test sets (5 × 8 = 40).

**Figure 6:**
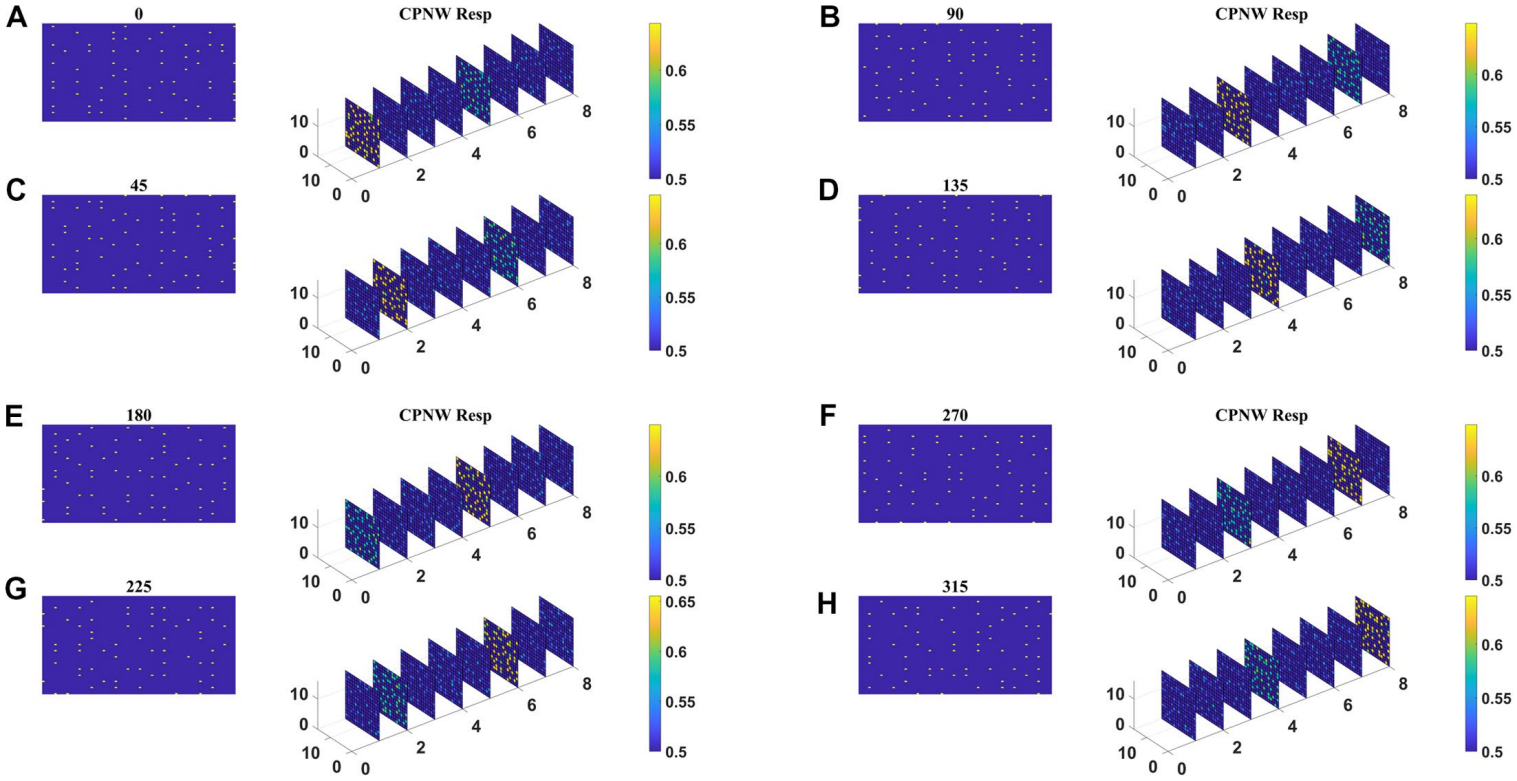
CPNW response to translational sequences: In all figures, **(A–H)** a frame (80 × 80) of a translational sequence and its corresponding response on CPNW is displayed. The numbers 0, 45, 90 etc., represent the direction of motion of the input sequence. CPNW consists of eight cell-planes, each shows maximum response to specific translational motion direction as result of training. The amount of activity produced by cell-plane neurons can be estimated using color bars. One can also observe that, though only one cell-plane produces highest activity in response to given motion direction (e.g., 0°), the cell-plane that encode opposite motion direction (180°) produce relatively high activity compared to the cell-planes that encode other motion directions.

The responses of the network stabilize after the training for 1,000 epochs. Before the testing phase, the training set is presented to the CPNW, and the winning cell plane for each translational direction is recorded and used as a label to estimate CPNW performance on the test set. Eight different cell planes showed maximum responses to 8 different translational motion directions provided in the input stimuli. Now we present the test set consisting of eight translational motions of eight different directions (0, 45, 90, 135, 180, 225, 270, and 315°) that are not seen by the network before. As shown in Figures 6A–H, each of the test sequences was responded to maximally and uniquely by one of the eight cell planes. We see that though each cell plane responded most strongly to its preferred translational motion directions, it also responded to the neighboring directions with lesser intensity. CPNW showed 100% accuracy on the test set.

#### Three-stage network response/perceptron response to optic flow sequence

**Figure 7:**
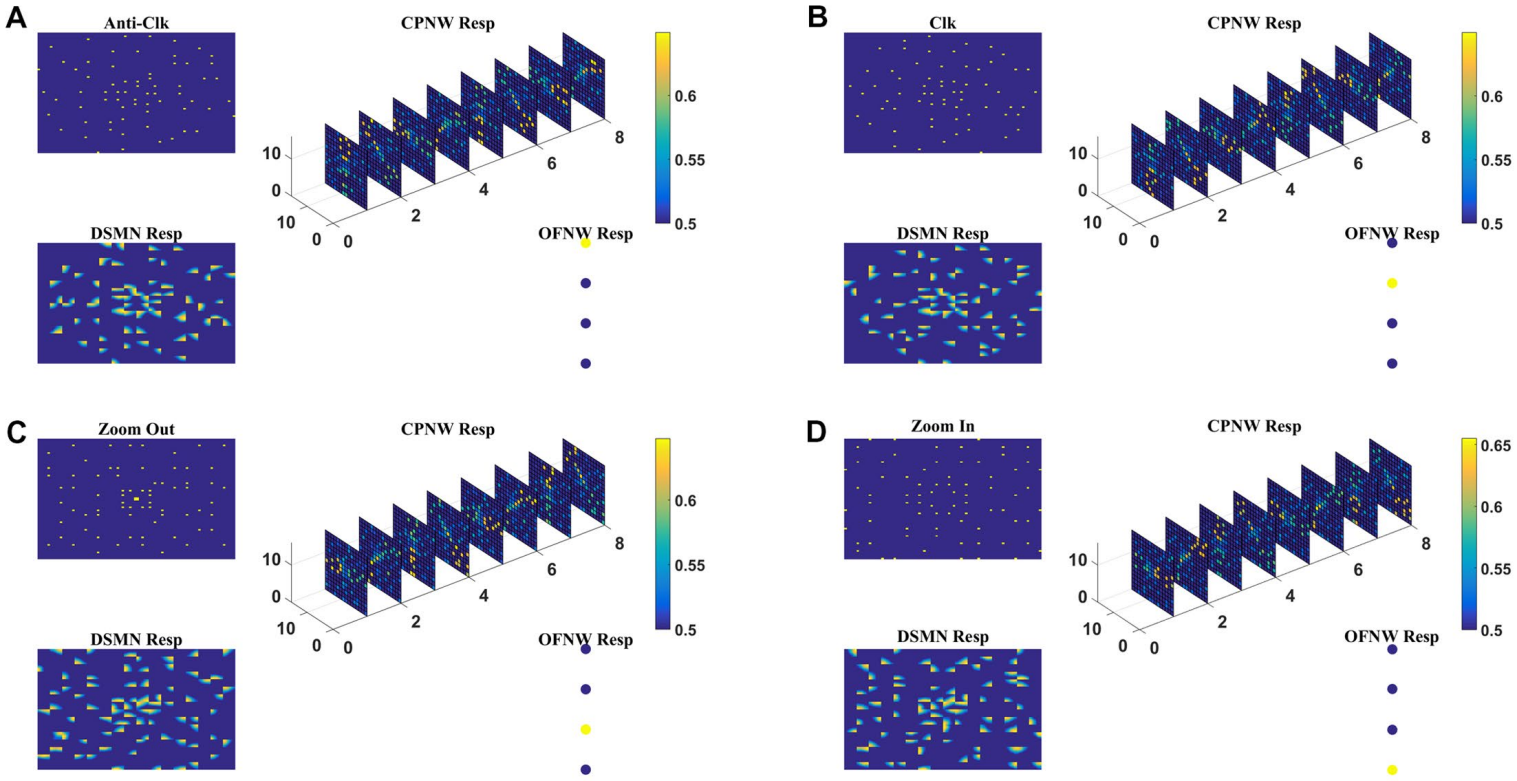
The response of Model-1 to optic flow sequences. The response of OFNW to **(A)** Anti-clockwise sequence, **(B)** clockwise sequence, **(C)** zooms out or radially outward sequence, and **(D)** zoom in or radially inward sequence. In each case, “DSMN Resp” represents the populations of neurons that are active in each tile of DSMN. “CPNW Resp” represents the subset of neurons that are active in each cell-plane in response to the given optic flow stimulus. The responses on eight cell-planes are arranged as a 1D vector before giving it to OFNW. OFNW is a four-class perceptron made up of two layers (input and output), and its response to four types of optic flow patterns is shown as OFNW Resp.

Here we train the optic flow network (multi-class perceptron) simulating MST neurons and test the complete model-1 composed of all three stages: initial DSMN, middle CPNW, and output OFNW/ perceptron. The training set to train OFNW/ perceptron is composed of optic flow sequences including contraction, expansion, clockwise rotation, and counterclockwise rotation each with 15 initial dot positions distributed over the image space. Thus, the training set is made up of 40 (10 × 4) flow sequences (see subsection optic flow motion in 2.10). We did not include planar translational patterns because here the main concern was the network’s response to different types of flow motion. The OFNW/ perceptron is trained by repeatedly presenting sequences in the training set in a random order to the DSMN. While training the perceptron, the weights of DSMN and CPNW that were trained earlier were kept constant and only their responses were fed forward to OFNW/perceptron. After 500 epochs, stable responses were obtained in OFNW/ perceptron. Now the test set comprising 20 sequences (5 initial dots x 4 flow types) is presented to the three-stage network (model-1) and the response is plotted in Figure 7. Figures 7A–D, respectively, show the model-1 response to anti-clockwise, clockwise, expansion (Zoom Out), and contraction (Zoom In) motions.

### Model-2

#### Two-stage network response/HBNW response to translational motion

The first stage in model-2 is DSMN, which is the same as in model-1. The second stage, HBNW, is made up of a 16 × 16 × 8 array of neurons. The training set used to train the CPNW in model-1 is now used to train the HBNW. As a result of training, the neurons in the HBNW learn to encode the local flow direction present in the small part of the image and on the whole, continuum of neurons (16 × 16 × 8) encodes global motion information present in the input sequence. HBNW is trained by repeatedly presenting translational motion sequences to DSMN, whose responses in turn were forwarded to HBNW neurons. Training is carried out for 10,000 epochs (learning rate = 0.05).

**Figure 8:**
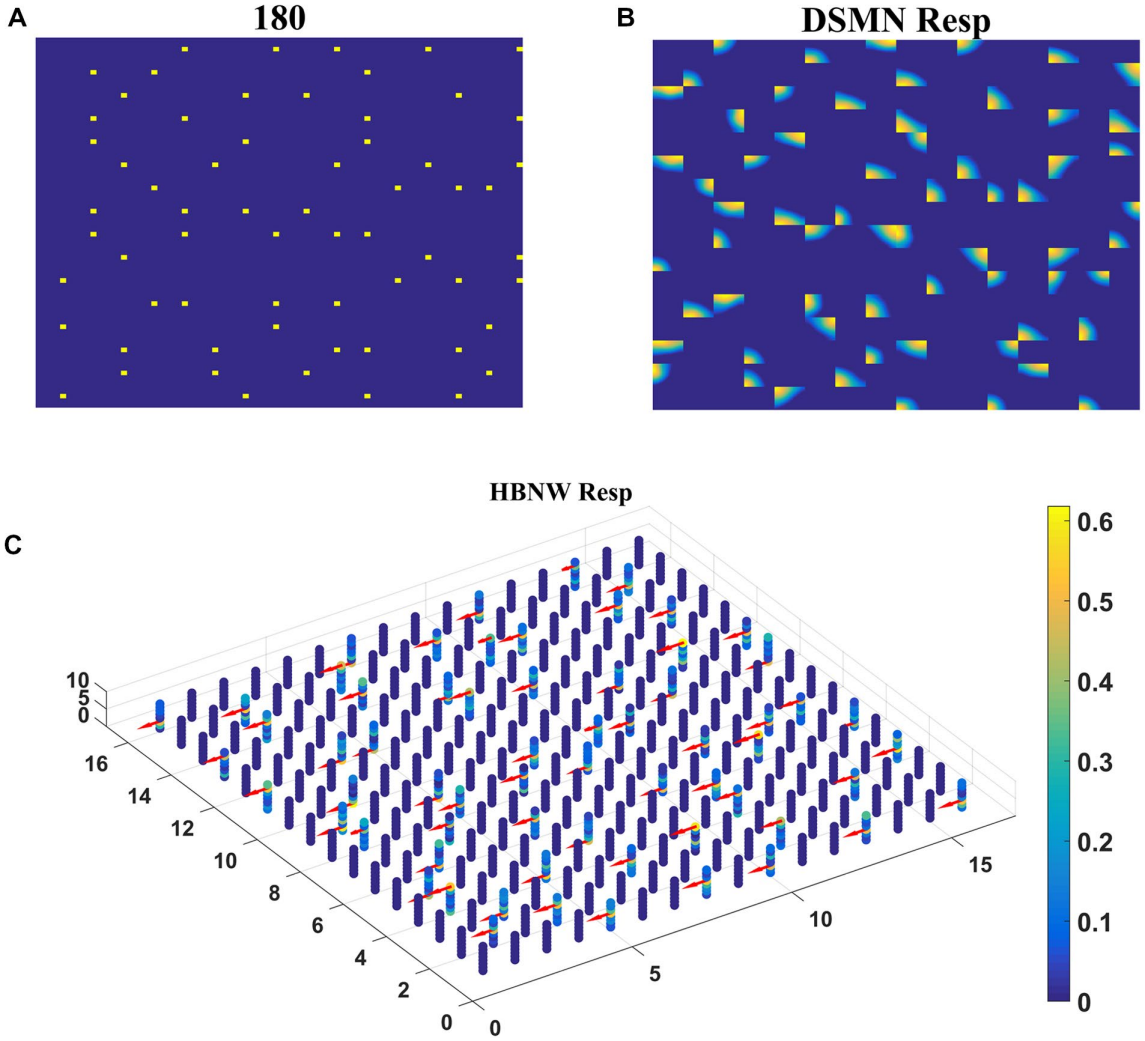
HBNW response to translational motion sequences. Here we plotted HBNW response to translational motion sequence: 180°. **(A)** represents a frame in an input sequence and corresponding DSMN response is shown in **(B)**. Hebbian network (16 × 16 × 8) response titled as “HBNW Resp” is plotted in **(C)**. At each vertical column, the winner is highlighted with the arrow whose head indicate the neuron’s direction preference.

Trained HBNW responses to translational sequences of 180° are plotted in Figure 8. Figure 8A displays the first frame of the translational motion sequence, Figures 8B,C represent the corresponding DSMN and HBNW responses, respectively. In Figure 8C, one can observe that, at each (*m*, *n*) location along z direction, only one neuron shows the highest response (winner) ~0.6 as indicated in the color bar; also, the winners at each vertical column are quite distinct. This is because a group of neurons along each vertical column that takes the same DSMN tile response will be trained to recognize salient motion direction existing in the input pattern in the sense that a similar set of input patterns (i.e., patterns having the same translational motion direction) will always excite one particular neuron and inhibit all others. Thus, during the training process, due to initial random afferent connections, one neuron from the group (m, n) shows the highest response, becomes a winner, and gets its weights updated. During training, other neurons within the group will become winners when they encounter a different input. At the end of the training, all eight neurons within a group get tuned to eight different directions. The arrows are plotted in Figure 8C at each winner neuron to indicate their direction preferences. By the end of this competitive learning process, the continuum of input patterns is divided into a set of clusters, each cluster is represented by a particular population of responding neurons. Note that each input pattern activates a large number of HBNW neurons, but the response selectivity is represented by the population of winner neurons. Thus, the different motion patterns presented to the two-stage network form a set of separable clusters in the feature space, which is typical for competitive learning. However, the class boundaries are not as crisp as in the case of CPNW.

**Figure 9:**
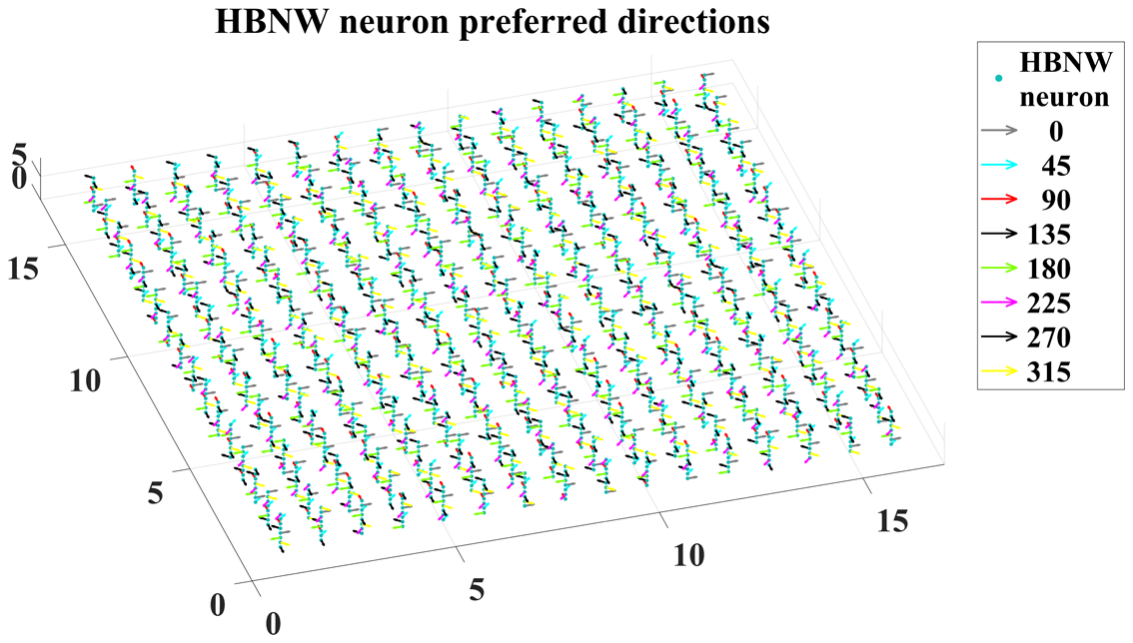
It shows the direction preferences developed by HBNW neurons in response to training (can be interpreted by observing colored arrows more carefully).

Trained HBNW neurons show the highest response when their preferred direction best fits the local motion direction in the input. We observed the winner neuron responses by presenting all 120 translational motion stimuli. Eight winner neurons were observed in response to 8 motion directions along each vertical column. The direction preferences of HBNW neurons are plotted in Figure 9. One can compare the winner neurons shown in Figure 8C with the neuron preferences shown in Figure 9. We observed that the HBNW neuron in each vertical column encodes eight different motion directions, without allowing the emergence of redundant and dead neurons.

#### Three-stage network response/MLP response to optic flow motion

Here we train the OFNW (multi-layer perceptron) simulating MST neurons and test model-2 composed of all three stages: DSMN, HBNW, and OFNW/MLP. The MLP consists of an input layer (2048 × 1), three hidden layers each of size 256 × 1, 156 × 1, and 50 × 1, and an output layer (4 × 1; Figure 3). It is trained using regular backpropagation algorithm for 5,000 epochs (learning rate = 0.1). The activation function used by nodes in the hidden and output layers is sigmoid and SoftMax, respectively. Note that the neuron responses produced by CPNW are very different from the responses produced in HBNW during competitive learning.

**Figure 10:**
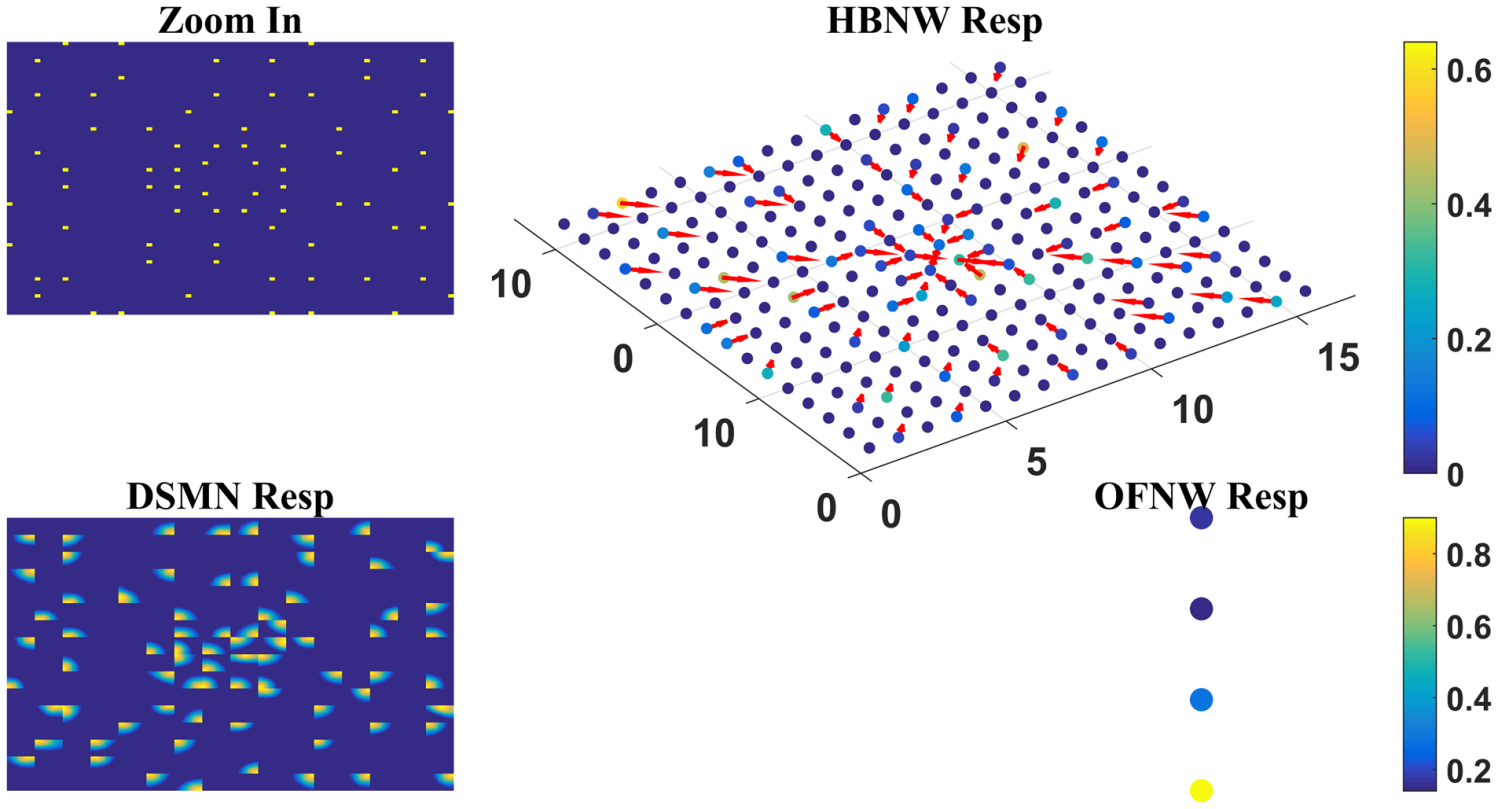
Model-2 responses to a “Zoom In” optic flow sequence. “Zoom In” represents frame in a radially inward flow. “DSMN Resp” represent the populations of neurons that are active in each tile to the input sequence. Hebbian network response (top view) is plotted under “HBNW Resp.” Each column is represented by a winner along with its direction preference (arrow). “OFNW Resp” represents the response of the classification layer nodes in MLP, each node is encoding specific flow type.

As the nature of the input presented to the output stage varies in model-1 and 2, different classification algorithms were proposed for OFNW. Similar to model-1 the training and test set for MLP in model-2 is composed of 40 and 20 flow sequences, respectively, each including contraction, expansion, clockwise rotation, and anti-clockwise rotation. While training MLP, DSMN, and HBNW weights were kept constant. The accuracy obtained on a training set and test set is 100 and 95%, respectively. MLP recognized one anti-clockwise motion sequence in the test set as a clockwise one. Once MLP training is completed, the response of the three-stage network is observed for every sequence in the test set. The response for one “Zoom In” sequence is plotted in Figure 10 where “OFNW resp” represents the activity of the output layer neurons in MLP.

At this stage, we would like to discuss why we used MLP in model-2 whereas perceptron in model-1. The second stage of model-1 is a CPNW where each cell plane is trained independently with dots translating in a specific direction. As a result, each of the test sequences is encoded maximally by one of the eight cell planes, even though some activity is produced by other cell planes as shown in Figure 6. Thus, the data set created using CPNW responses to train third stage OFNW is linearly separable. Linearly separable data can be encoded by a simple perceptron. On the other hand, model-2 s stage consists of HBNW. In HBNW, a group of neurons along each vertical column takes the same DSMN tile response, and are trained to recognize salient motion direction existing in the input pattern through competitive learning. In other words, different neurons at different vertical columns (neurons highlighted with arrows in Figure 8C) have become winners and on the whole different continuum of neurons encode different translational motion directions. Thus, the data set created with HBNW responses are highly non-linear and can be encoded by only networks like multi-layer perceptron’s.

### Model-3

Model-2 is a biologically plausible optic flow recognition model, in which training of initial, middle, and output stages is designed based on the knowledge of the response properties of the neurons present at various levels of motion pathway. On the other hand, in recent years, data-driven CNNs, developed based on early studies of the visual system (Schmidhuber, 2015), have been widely used as visual encoding models. These encoding models work by establishing a nonlinear mapping from visual stimuli to features, and a suitable feature transformation is critical for the encoding performance (Tang et al., 2018). Studies have also shown that a deep network is comparable to the human visual system, which can automatically learn salient features from large data for specific tasks (Agrawal et al., 2014; Cohen et al., 2017). Recent studies by Güçlü and van Gerven demonstrated the similarity between CNN and visual pathways, revealing a complexity gradient from lower layers to the higher layers (Güçlü and van Gerven, 2015, 2017). Along similar lines, we developed a data-driven optic flow recognition model (presented in Figure 1D) using CNNs.

#### VSMN tile response

In this study, we investigate whether CNN can serve as a model of the macaque motion-processing network. There is evidence that, unlike V1 neurons, a subset of neurons within the primate extrastriate cortex (MT) appear broadly tuned to local image speed and direction (Movshon et al., 1985; Maunsell and Newsome, 1987). The model is said to be biologically plausible if and only if V1, MT, and MST representative neurons in the model show response properties analogous to those of real V1, MT, and MST neurons. Currently, the middle stage (CPNW, HBNW) neurons in model-1 and model-2 estimate local flow motion purely based on the direction-selective responses from DSMN. To simulate MT-like responses to the local image motion according to the product of their direction and speed responses, we created Velocity Selective Mosaic Network (VSMN) in place of DSMN, which encodes speed together with direction.

**Figure 11:**
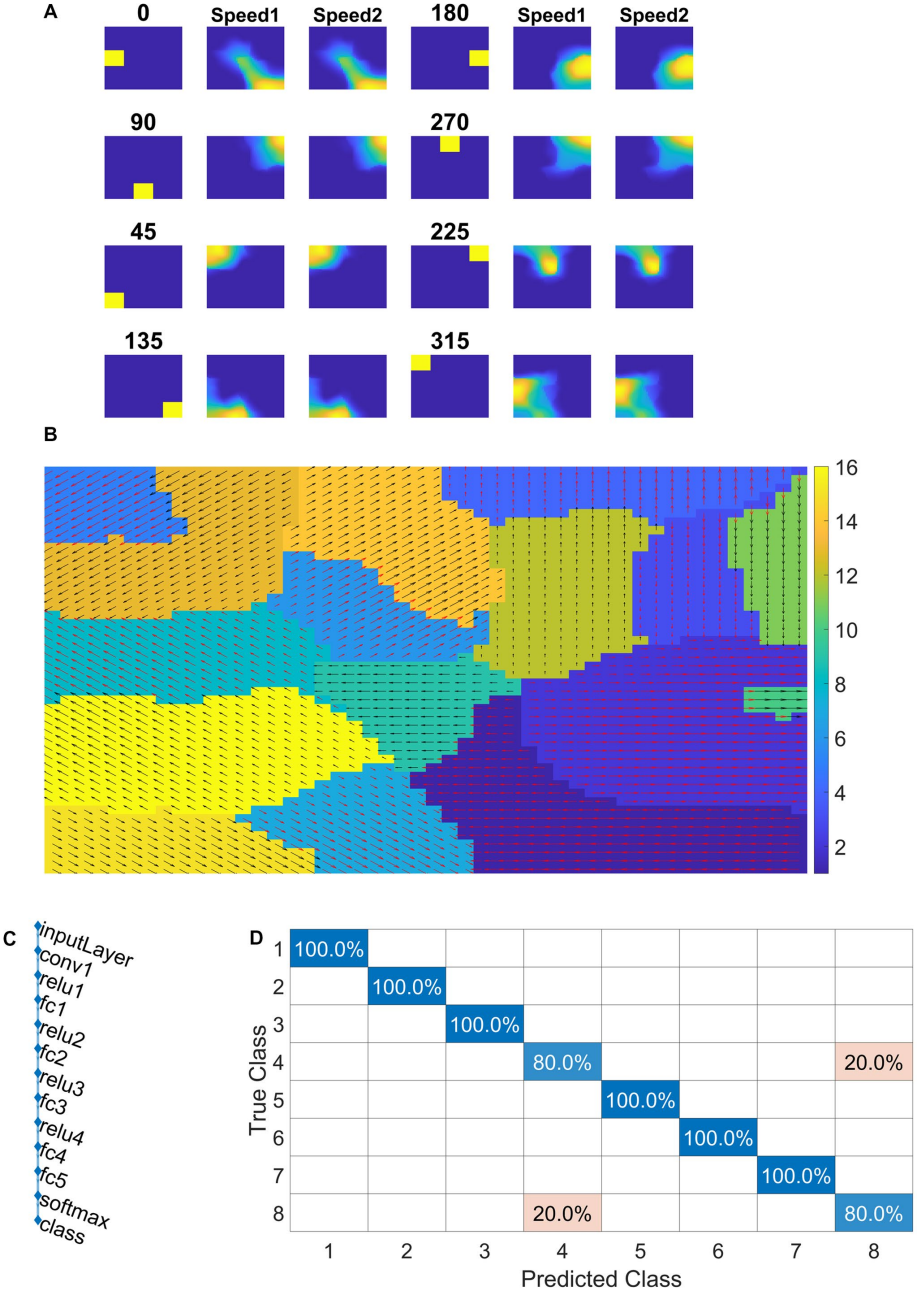
NF response in VSMN. In **(A)** first and fourth columns display the first frame (8 × 8) of an input sequence that consists of dots (2 × 2) moving in specific direction with two speeds. The second, third, fifth, and sixth columns represent the corresponding NF (48 × 48) activity. The same neuron population become selective to the inputs of same direction and different speeds, however within the population neurons have different speed preferences which are shown in **(B)** the velocity selective map. Neuron direction preferences are shown by head of the arrow and the speed preferences are shown by the arrow color. Color bars indicate 16 input types (8 directions × 2 speeds). 1–8 belong to speed-1 and 9–16 to speed-2. **(C)** shows the architecture of CNN. **(D)** shows the confusion matrix obtained for the test set.

Model-3 consists of VSMN followed by CNN (Figure 4). Each tile in VSMN is an NF and is trained to recognize the velocity of the input stimuli as described in section “Velocity selective mosaic network”. Figure 11A shows the response of a tile in VSMN for the input stimuli consisting of 2 × 2 dots moving in eight directions. Here dot is allowed to move one pixel and two pixels ahead for each time step constitutes two speeds. Thus, input stimuli consist of 16 inputs (eight directions and two speeds). Figure 11B plots the velocity selective map consisting of 16 populations. Each population is highlighted with a colored arrow (red for speed1 and black for speed2), indicating the preferred speed and direction of the motion.

#### Training CNN with optic flow sequences

Convolutional neural network is trained with eight classes. Thus, the training stimuli for CNN are made up of four types of flow sequences (expansion, contraction, clockwise rotation, and anti-clockwise rotation) and each occurs at two speeds. A total of 80 motion sequences (10 initial dot positions × 4 flow types × 2 speeds) were generated for the training set and 40 (5 × 4 × 2) sequences for the test set. The CNN is trained to recognize the type of optic flow present in a given input sequence, by repeatedly and randomly presenting input from the training set to its previous VSMN, whose final response is forwarded to the CNN. The architecture of the CNN and various learning parameters used to train the CNN are described in Table 2. Note that the CNN is trained only on optic flow stimuli and not exposed to translational stimuli. Figures 11C,D indicate the CNN architecture, the confusion matrix, and the learning curve, respectively. For the implementation of CNN and to visualize the CNN feature maps, we used MATLAB with Deep Learning Toolbox. The trained CNN gave classification accuracy—100% for training data and 90% for held-out test data.

**Table 2:** CNN architecture and learning parameters used.

Layer	Size
Convolution Layer (conv1)	48 × 48 × 36
Fully connected layer (fc1)	350 × 1
Fully connected layer (fc2)	150 × 1
Fully connected layer (fc3)	150 × 1
Fully connected layer (fc4)	100 × 1
Output layer	8 × 1
Learning parameters	
Learning rate	0.01
Batch size	20
Epochs	350

#### CNN response to translational and flow sequences

**Figure 12:**
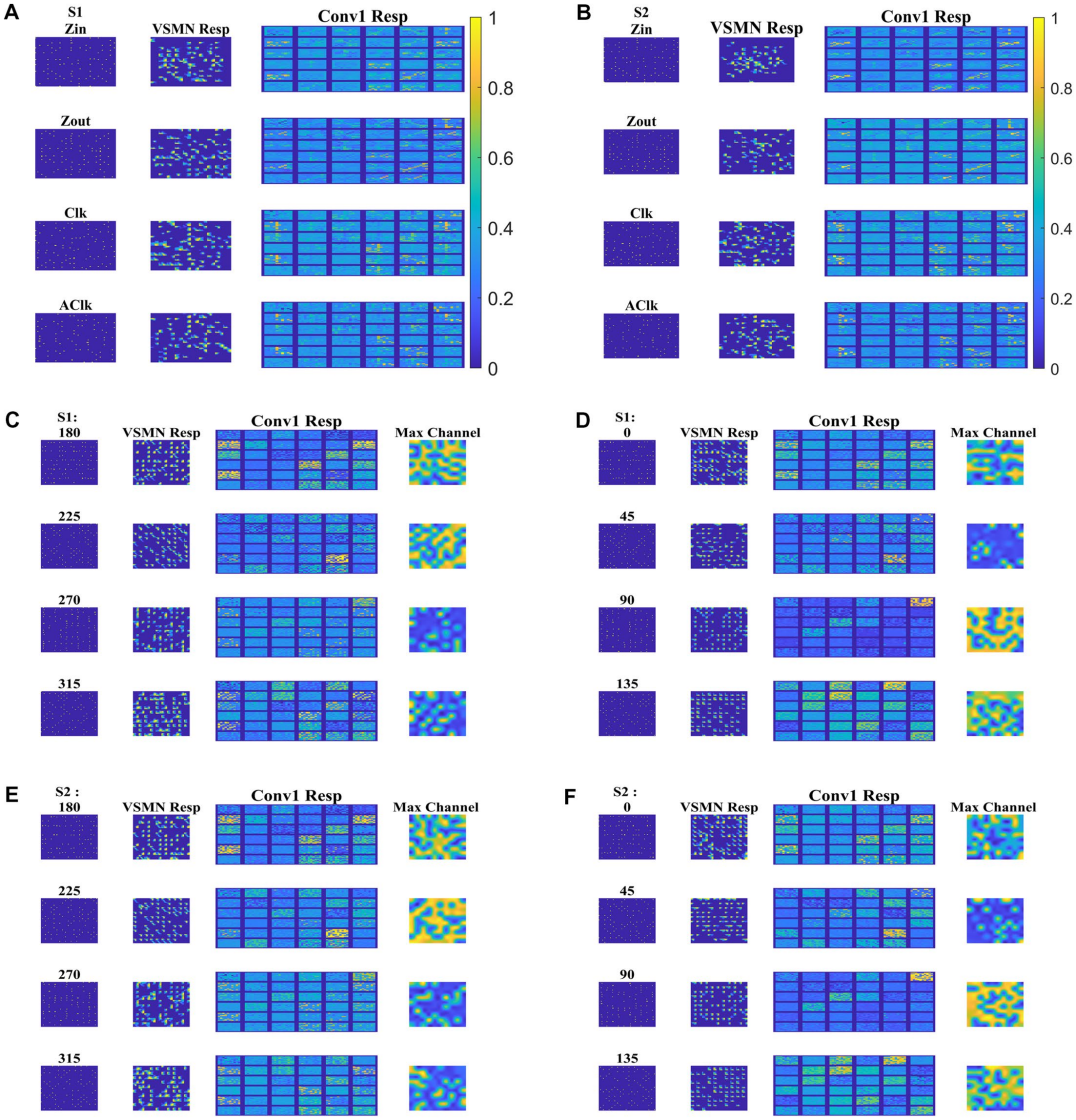
Model-3 responses to optic flow and translational motion sequences. In **(A,B)**, first and second columns display the frame of an optic flow sequence and the corresponding VSMN response, respectively. “Conv1 Resp”—indicates the activity on 36 feature maps of convolution layer. In **(C–F)**, first, second, and third columns display the frame of a translational sequence, corresponding VSMN response, and the convolution layer activity, respectively. Out of 36 feature maps, the channel with the maximum response is plotted in the fourth column as “Max Channel.”

Next, the trained CNN is presented with both translational and optic flow sequences, and the responses are plotted in Figure 12. “Conv1” layer consists of 36 feature maps (Figure 4). All these feature maps are arranged in a grid-like structure and plotted as shown in “Conv1 Resp.” Figures 12A,B plots the conv1 response to four types of optic flow stimuli moving with speed-1 and speed-2, respectively. Whereas Figures 12C–F display the conv1 response to translational stimuli moving eight directions with speed-1 and speed-2, respectively. Note that the CNN network was never exposed to translational patterns during training. Still diffuse and sparse response patterns could be observed under the conv1 response column, in response to translational and flow sequences, respectively. One can compare these responses with the responses of CPNW, as shown in Figures 6, 7, where we obtained similar diffuse responses to translational sequences and sparse responses to optic flow sequences.

#### Development of translational motion selectivity and speed selectivity in conv1

**Figure 13:**
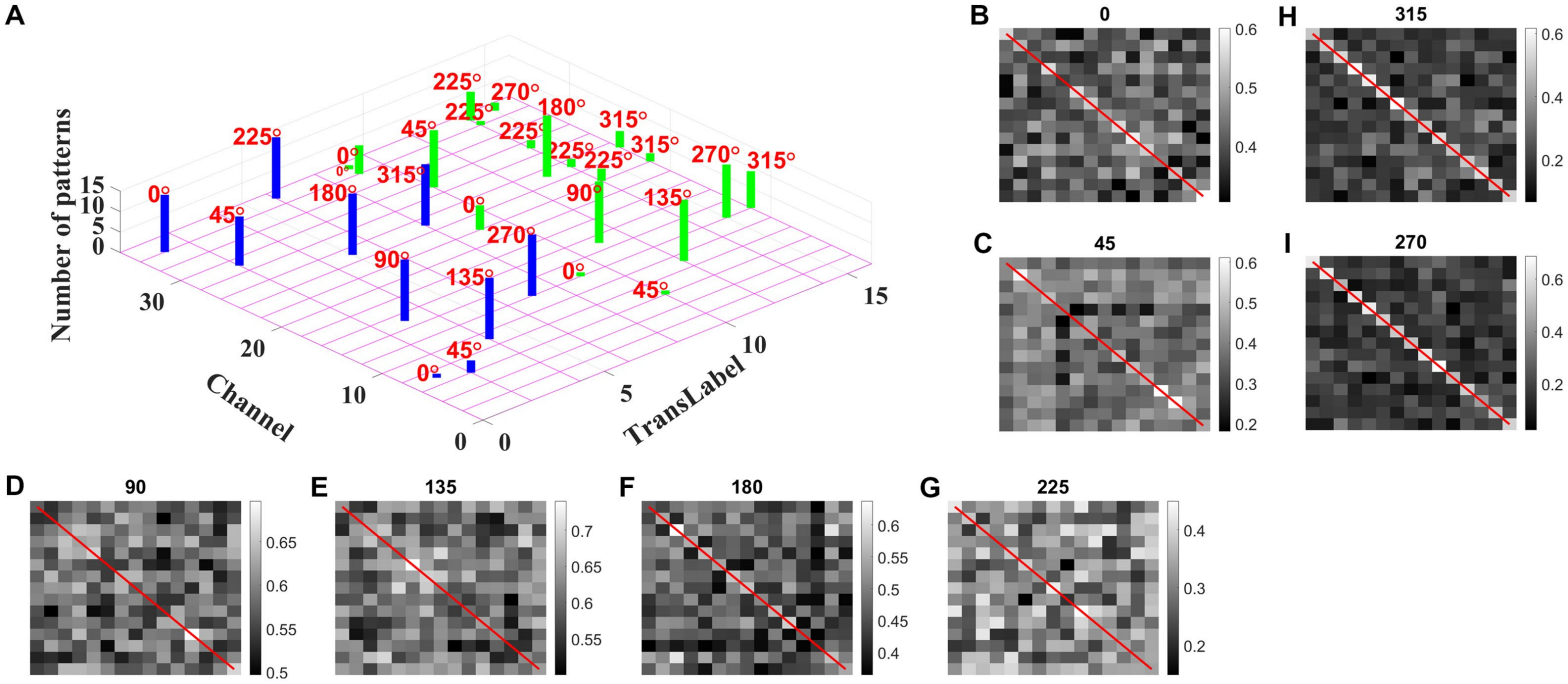
Development of speed selectivity in Conv1 layer. Each bar in **(A)** indicates the active channel corresponding to each input class (16 classes = 8 directions × 2 speeds; test set consists of 240 sequences: 15 initial dot positions × 16 classes; labels 1–8 belong to speed-1 inputs and are indicated by blue bars; labels 9–16 belong to speed-2 inputs and are indicated by green bars). For example, 0° translational sequence with speed-1 activates two channels 7 and 34. However, more inputs activate channel 34 as indicated by bar length. One can observe that different channels encode different motion directions. However, inputs moving in same direction with different speeds seems to activate the same channel in bar graph. Speed selectivity in Conv1 layer using RSMs. **(B–I)** represent the response similarity matrices (RSMs). The matrix entries (15 × 15) indicate the pairwise correlation coefficients for speed-1 and speed-2 translational motion sequences whose direction is indicated on top of each figure. The diagonal elements of a matrix (as highlighted with red line) represent correlation coefficient calculated for the pair of sequences having same initial dot configuration and moving with different speeds. Most of the RSMs have no strong block diagonal structure (except for 315 and 270°), indicating that different neuronal populations in conv1 respond to different speeds. For 315 and 270° block diagonal structure can be seen due to the very low correlation values, not because of similar responses of conv1 channel to both speeds, as the high correlation value is 0.6 as indicated in color bar. Thus, even though the same channel codes for a sequence with different speeds, within the channel different subpopulations code for different speeds.

We may now ask whether the CNN trained on optic flow motion sequences developed selectivity to translational sequences in its lower conv1 layer. If that is the case, the conv1 layer is analogous to MT and CNN’s output layer is analogous to MST. To verify the above hypothesis, we presented the trained network with a test set, consisting of 240 translational sequences moving in eight directions, with two speeds, and started from 15 different initial dot configurations (15 × 8 × 2 = 16 classes with 15 sequences in each class). For all 240 inputs, the conv1 feature map or a channel with a maximum response is noted and the bar graph against each class is displayed, as shown in Figure 13A.

As shown in Figure 13A, different channels respond maximally to different translational directions, and also more than one channel showed the highest activity in the same motion direction. These results are consistent with previous reports of a high degree of direction selectivity in MT with nearby units having similar preferred directions (Maunsell and van Essen, 1983a). These studies also reported that the MT units were sharply tuned for the speed of stimulus motion. However, the same channel in the conv1 layer seems to become highly active to the inputs with the same translational direction but moving at different speeds (as indicated by bars: blue-speed-1 and green-speed-2). MT neurons must have different speed characteristics to be consistent with the MT studies (Lagae et al., 1993). Orban et al. (1981) grouped cells in areas 17 and 18 into four distinct classes based on the broadness of the speed tuning curve and the speeds to which they responded. To understand whether the conv1 layer developed speed selectivity as a result of training, we computed correlation matrices and plotted them, as shown in Figures 13B–I. Initially, conv1 responses for all 240 translational inputs are obtained. For each direction, the Pearson correlation between each pair of speed-1 and speed-2 (15 × 15 pairs) is calculated. The diagonal elements of a matrix (as highlighted with a red line) represent the correlation coefficient measured between the translational patterns moving at two different speeds (speed-1 and speed-2) starting with the same initial dot configuration. Smaller correlation values, as shown by the color bar and lack of block diagonal structure in plots (B–I), indicate that conv1 neurons display different responses to different speed stimuli, which is consistent with the cell properties in MT (Graziano, 1990; Duffy and Wurtz, 1991a,b, 1995; Graziano et al., 1994; Duffy, 1998). Further to quantify this speed selectivity by conv1 channel, we trained a two-class linear classifier for each case displayed in plots (B-I). The accuracies obtained on the test set for each direction 0, 45, 90, 135, 180, 225, 270, and 315° are 80, 70, 90, 80, 80, 70, 90, and 100%, respectively. Thus, when CNN is trained using optic flow motion stimuli, the lower layers develop selectivity to translational motion which is analogous to MT.

**Figure 14:**
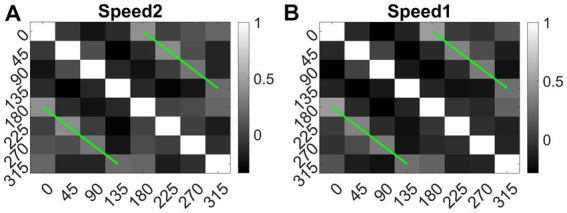
Direction tuning in conv1 layer. **(A, B)** shows conv1 response similarity matrix (8 × 8) for speed1 and speed2 respectively. Entries in the matrix indicating pairwise Euclidean distance between responses to translational motion directions (one sequence considered for each direction). The directions are indicated along the axes. From the plot one can observe that the conv1 layer produce similar response to the sequences with opposite directions. Matrix entries along green lines are positively correlated and the entries between orthogonal angle are negatively correlated (values are <0).

We also calculated the Euclidean distance between conv1 responses to each pair of eight translational directions, and for each speed separately. As shown in Figure 14 (green lines), the responses to dot patterns moving in opposite directions (0–180, 45–225, 135–315, and 90–270°) have a higher correlation compared to the other pairs. Similar responses were seen while comparing the responses to the speed-1 and speed-2 stimuli set. Thus, translational patterns with opposite motion directions develop similar activity patterns on the conv1 layer, suggesting that they are closer in feature space.

#### Correlating CNN responses with known properties of the motion processing hierarchy

To investigate whether the CNN can explain known tuning properties of the macaque motion processing network (Maunsell and van Essen, 1983a,b; Graziano, 1990; Duffy and Wurtz, 1991a,b, 1995; Graziano et al., 1994), we calculated the responses of CNN layers (conv1and fc4) to translational (240) and flow (120) sequences made out of training together with test sets. We then calculated the correlation between the population responses to each pair of these sequences and constructed a population Response Similarity Matrix (RSM) of such correlations for all pairs.

**Figure 15:**
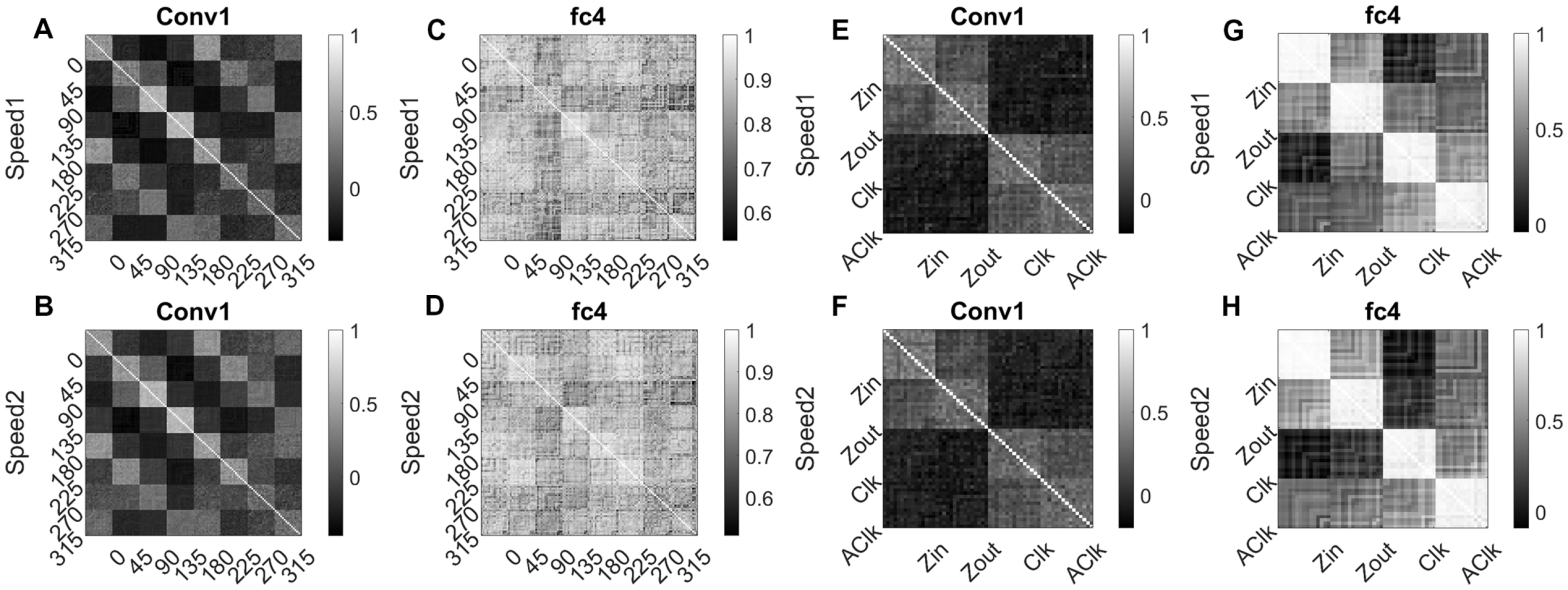
Correlating Conv1 responses with the cell responses in motion hierarchy. Plots display RSMs for CNN layer (conv1) and last hidden layer (fc4). Matrix entries in **(A–D)** indicate the pairwise correlation coefficients calculated for the translational responses, separately for each speed. Matrix entries in **(E–H)** indicate the pairwise correlation coefficients for the optic flow motion responses, separately for each speed. Elements of the matrices are grouped according to the direction and flow type as indicated along the axes.

Figure 15 shows RSMs for the convolution layer (conv1) and last hidden layer (fc4), for both translational motion and optic flow motion. In Figures 15A–D, the translational sequence numbers are grouped according to the motion direction (each forming sub-matrices of size 15 × 15). In Figures 15E–H, the optic flow motion class numbers represent the type of the flow (here also each sub-matrix is of size 15 × 15). Each of the 15 × 15 response similarity matrices indicates the pairwise correlation coefficients calculated for sequences made out of 15 initial dot configurations. In the conv1 layer, the RSMs corresponding to the translational stimuli (Figures 15A,B) show that different conv1 neuronal populations show selectivity to a different translational motion direction. In Figures 15A,B, the strong block-diagonal patterns can be seen, indicating that the populations show selectivity to a specific direction of motion irrespective of the initial dot position. Also, RSM entries between opposite motion directions are positively correlated, whereas RSM entries between orthogonal pairs are negatively correlated, indicating that opposite motion directions at the input space are arranged more closely at the feature space than the orthogonal motion directions. Figures 15C,D display the RSMs corresponding to the translational stimuli in layer fc4 (the last hidden layer). Even though a weak block-diagonal pattern can be seen, the clear distinct response profiles to various translational directions were not seen. It appears neurons in layer fc4 do not have specific selectivity to translational motion direction; they instead respond to all translational directions more or less equally.

Figures 15E,F display RSMs corresponding to optic flow stimuli in the conv1 layer, showing a clear distinction between radial and circular motion types, with less prominent selectivity to specific flow types. Whereas, as shown in Figures 15G,H, very strong block-diagonal along with distinct RSM entries of the fc4 layer, indicating that the units are highly selective to different flow types. To understand more about the above conv1 and fc4 responses and their selectivity to various types of translational and flow sequences, we built and trained a linear classifier. In case-1where perceptron trained with “conv1 responses to translational stimuli” produced good recognition accuracy on the test set in both cases: 85% in speed-1 and 87% in speed-2. In case-2 where perceptron trained with “fc4 responses to translational stimuli” produced less recognition accuracy on the test set in both cases: 37% in speed-1 and 48% in speed-2. In case-3 where perceptron trained with “fc4 responses to optic flow stimuli” produced high accuracy on the test set (100% in both cases), However, in Case-4 where perceptron is trained on “conv1 responses to optic flow stimuli” produced high accuracy (95% in both cases). It appears that lower layers in the CNN develop selectivity to translational motion while the higher layers code for only the optic flow motion. In sum, the bottom-to-top layers of model-3 gradually shifted from direction selectivity of V1 cells to local flow motion estimation and finally optic flow type selectivity, which is reasonably consistent with the idea of functional hierarchy in the macaque motion processing (Hubel, 1988; Duffy and Wurtz, 1991a,b; Born and Bradley, 2005).

## Discussion

Over the last decades, vision research had unraveled a cascade of motion processing stages within the hierarchy of visual cortical areas (V1-MT-MSTd) along the dorsal pathway. V1 neurons have small receptive fields (~0.5–2°; Zeki, 1993; Duffy and Hubel, 2007) and therefore can analyze movement over only a tiny portion of the visual field. The extra-striate middle temporal area (MT or V5) has a receptive field 10 times the size of V1, while still covering only a relatively small fraction (~2–15°; Saito et al., 1986; Komatsu and Wurtz, 1988a; Richert et al., 2013) of the visual field. However, optic flow covers the entire visual field. The medial superior temporal area (MSTd) has receptive fields (>30°; Raiguel et al., 1997; Amano et al., 2009) that cover large parts of the visual field (Saito et al., 1986; Komatsu and Wurtz, 1988b) are said to be coding for optic flow motion. The three models described in this paper were based on this functional hierarchy and learn to recognize the type of optic flow present in the given dot sequence.

Here first we list out some of the key features of experimental MSTd cell responses (Graziano, 1990; Duffy and Wurtz, 1991a,b, 1995; Graziano et al., 1994), to interpret the performance of the proposed models. (i) The receptive fields of MSTd cells are (> 30°) much larger than those of the MT cells. (ii) MSTd cells respond to different types of motion stimuli such as unidirectional planar/translational motion, clockwise and counter-clockwise rotational motion, outward and inward radial motion, and various spiral motions (Graziano, 1990; Duffy and Wurtz, 1991a,b; Graziano et al., 1994). (iii) Some MSTd neurons respond maximally to the specific motion type and less strongly, to neighboring motion types (Duffy and Wurtz, 1995), while others respond moderately to all motion types. Some neurons do not respond selectively to any of the motion components (Duffy and Wurtz, 1991a; Graziano et al., 1994).

Based on the results provided in Sections 3.1 and 3.2, we can summarize the response properties of neurons in model-1 and model-2 as follows. (i) The neurons in the OFNW simulating MSTd neurons have much larger receptive fields than the neurons in DSMN. Also, they respond to different types of optic flow motion stimuli, including radial and circular motions. (ii) Many neurons in CPNW and HBNW respond most strongly to their preferred translational motion direction, while they also respond less strongly to neighboring directions and moderately to opposite directions. These features match well with the properties of experimental MSTd neurons.

However, neurons in MT that respond to different translational motion directions form a continuum of response selectivities instead of discrete classes (Recanzone et al., 1997) similar to the case with MSTd neurons. In CPNW, since there is no competition across cell planes, neurons form a discrete class with different directional preferences (i.e., all the neurons in one cell plane own the same direction preference, and neurons in different cell planes exhibit different direction preferences), which is not in agreement with properties of neurons found experimentally (Duffy and Wurtz, 1991a; Graziano et al., 1994; Recanzone et al., 1997). We developed the Hebbian network (HBNW) by incorporating competition across the neurons as described in Section 2.4. Through competitive learning, HBNW neurons along each vertical column are trained to respond selectively to the different motion directions, which form a continuum of response selectivity along a 2D array of neurons. This continuum of responses divided, relatively evenly and randomly, into a set of clusters, each represented by a particular output neuron in OFNW, which is consistent with the empirical findings (Duffy and Wurtz, 1991a; Graziano et al., 1994; Recanzone et al., 1997). Thus, for the presentation of each class of translational motion sequence, the two-stage network (DSMN + HBNW) exhibits a sparsely distributed set of active neurons that represents the translation direction of the feature space, which is typical for competitive learning.

Also, HBNW winners corresponding to each input flow sequence are arranged by the pattern of that optic flow type. In other words, as shown in Figure 10, “HBNW Resp” displays radial arrangement (winner preferences) for the presentation of radial motion. Similarly, a circular arrangement for the presentation of rotational motion. This is consistent with the hypothesis proposed by various researchers (Saito et al., 1986; Tanaka and Saito, 1989) that the receptive field of an MST cell responsive to circular or radial motions is composed of a set of directionally selective MT cells arranged by the pattern of that optic flow component.

The first two models presented in this paper simulate the direction-selective properties of V1 cells, the local flow selective responses of MT cells, and selective responses to various optic-flow motion types found in the MSTd area. Both these models show that sophisticated neuronal responses to motion stimuli can be accounted for by relatively simple network models. We also verified whether the first two models develop speed-selective responses when simulated with optic flow sequences having multiple speeds. We conducted experiments by replacing first stage of model-1 and model-2 with VSMN instead of DSMN. Simulation results indicate that model-1 and model-2 can recognize inputs with different speeds with an accuracy of 85 and 82.5%, respectively, (details are provided in the Supplementary material). On the other hand, recent deep convolution neural networks (CNNs) have emphasized the layer-wise quantitative similarity between convolutional neural networks (CNNs) and the primate visual ventral stream (Yamins and DiCarlo, 2016). However, whether such similarity holds for the motion-selective areas in the motion pathway, is not clear through the above studies.

In the studies with model-3, we investigate whether CNNs can reproduce the tuning properties observed in the visual motion areas of the macaque brain. We explore the correspondence between the trained model-3 CNN layers and the macaque motion areas by calculating RSMs. Note that we did not constrain model-3 to match neural data, instead by comparing RSMs corresponding to translational and flow motion sequences at the convolution layer (conv1) and last hidden layer (fc4), we showed that the top output layer is highly predictive of MSTd responses and the intermediate convolutional layer (conv1) is highly predictive of neural responses in MT, an intermediate motion area that provides the dominant cortical input to MSTd. The correlation results show that, in model-3, as one traverse from the input to the output layer, response selectivity gradually shifts from direction selectivity (in VSMN), to local flow selectivity (in conv1), to flow type selectivity (in fc4), which is consistent with the idea of functional hierarchy in the macaque motion pathway. Furthermore, these studies indicate that CNN, in combination with basic sequence processing capabilities offered by DSMN, can be used to build quantitative models of motion processing.

### Biological relevance of model-2

Neurons in the individual NFs of DSMN are designed to have center-surround lateral connectivity, where the lateral connections are trained by asymmetric Hebbian learning. All these centers surround lateral connections and afferent connections are adapted through the asymmetric Hebbian rule. In the asymmetric Hebbian learning rule, the correlation between the presynaptic state at the current time and the postsynaptic state at a later time is used. Correspondingly, while training HBNW the symmetric Hebbian rule is used (Hebb, 1949a, 1949b) wherein the pre-and postsynaptic states are considered at the same instant. If we combine this with the winner take all rule, post-synaptic neurons compete with each other and the neurons that produce the largest response become the winner. Only the winner’s afferent connections are adapted by Hebbian learning. The winner-takes-all rule used in HBNW facilitates competition across the neurons so that different neurons become selective to the different motion directions present in the input. The adaptable lateral connections, Hebbian rule, and winner take all mechanisms are biologically plausible and have been tested experimentally (Salzman and Newsome, 1994). Likewise, the initial afferent response of the neurons in DSMN is passed through the piecewise-linear sigmoid function, and in HBNW the responses are passed through the sigmoid function to make the response nonlinear. Experimental studies have shown that responses of cells in visual cortical areas show significant nonlinearities depending on spatiotemporal activity distribution and also such response nonlinearities have been demonstrated in the LGN and in area V1 and beyond (Williams and Shapley, 2007; Solomon et al., 2010).

### Comparison between model-2 and model-3

Model-2 follows a bottom-up approach where neurons in the lower stages are trained first followed by the higher stages in the hierarchy. The initial/first stage neurons are trained to recognize the direction of moving stimuli present within the receptive field; the middle stage neurons are trained with translational dot sequences to encode the direction of local flow; and the last/output stage neurons are trained using different optic flow type sequences. In each stage, training is designed based on the experimental response properties of the neurons present at various levels of the motion pathway. This manner of modeling requires not only the knowledge of neurobiological findings but also a good grasp of various neural modeling approaches.

On the other hand, deep learning models are completely data-driven, easy to design and training, and require very little pre-programming and domain knowledge. Moreover, various studies (Kriegeskorte et al., 2008; Kriegeskorte and Kievit, 2013; Mur et al., 2013; Yamins et al., 2013; Kriegeskorte, 2015b) demonstrated the parallels along the hierarchy between layers of CNN and the visual areas of ventral pathway. Model-3 is trained end-to-end directly using optic flow motion sequences without any human intervention. Simulation results showed that CNNs can explain the experimentally observed tuning properties of motion areas MT and MSTd and also exhibit representational similarity with motion areas.

### Limitations of the existing model

The scope of the current study is the simulation of computation performed by motion pathway neurons like MT and MST. As a result of training, the three models develop responses similar to the responses of motion pathway neurons. Also, model-1 model-2 are developed by keeping biological realism in view. Within the given scope, we could not see any limitations in the proposed models. However, model is not designed to take real-world scenarios/ videos as input. One can view this as the limitation of the current model. In all the simulations, the model is exposed only to the current frame and the history about the past frames is stored in form of lateral interactions. Lateral connections are one way in which networks can retain stimulus history, which differentiates them from other computer vision models where the optic flow extraction is done by considering a set of frames.

However, even though it is the out of the scope of the current study, it is interesting to study about the model’s adaptability and performance when exposed to real time videos, such as tracking of unmanned aerial vehicles (UAV). Some recent works (Yuan et al., 2020, 2022) describe the state-of-the-art tracking methods such as spatio-temporal context-aware model and self-supervised deep correlation tracking. Another interesting study by same authors (Shu et al., 2020) proposed adaptive weight part-based convolutional network for person re-identification. However, the current model is not designed to process real-word video data.

## Conclusion

In this paper, we simulated three models to recognize the type of optic flow present in the input sequence. All the models explain different functional properties of neurons present in the motion pathway. Further model-3 can be viewed as the candidate model to explain the different aspects of motion processing apart from optic flow. In the future, we further would like to simulate model-3 to understand other motion aspects such as structure from motion and recognition of biological motion.

## Data availability statement

The original contributions presented in the study are included in the article/Supplementary material, further inquiries can be directed to the corresponding author.

## Author contributions

AG was involved in designing the model, coding, running simulations, and manuscript preparation. VC was involved in designing the model and manuscript preparation. All authors contributed to the article and approved the submitted version.

## Conflict of interest

The authors declare that the research was conducted in the absence of any commercial or financial relationships that could be construed as a potential conflict of interest.

## Publisher’s note

All claims expressed in this article are solely those of the authors and do not necessarily represent those of their affiliated organizations, or those of the publisher, the editors and the reviewers. Any product that may be evaluated in this article, or claim that may be made by its manufacturer, is not guaranteed or endorsed by the publisher.

## Footnotes

^1^However, more recent studies have identified direction-sensitive cells as early as in the retina (Wyatt and Daw, 1975; Wei et al., 2011).

## Abbreviations

NF: Neural field
DSMN: Direction selective mosaic network
VSMN: Velocity selective mosaic network
CPNW: Cell plane network
HBNW: Hebbian network
RSM: Response similarity matrix
OFNW: Optic flow network
CNN: Convolutional neural network
