# Supplementary material for: Modeling the development of cortical responses in primate dorsal (“where”) pathway to optic flow using hierarchical neural field models
Source: Front Neurosci. 2023 May 22;17:1154252. doi: 10.3389/fnins.2023.1154252 (PMC10239834; doi:10.3389/fnins.2023.1154252)
Supplement: Supplementary file 1 [file Data_Sheet_1.PDF]

SUPPLEMENTARY DOCUMENT

We simulated model-1 and model-2 by replacing the first stage direction selective mosaic network (DSMN) with a velocity selective mosaic network (VSMN), to verify whether they develop speed-selective responses or not.

1. Model-1

1.1. Two-stage network response/ *CPNW response to translated motion*

Here cell plane network (CPNW) is trained with responses of VSMN. We maintained 8 cell planes and are trained independently by repeatedly presenting translational motion sequences to VSMN. Each cell plane is trained with 20 dot sequences translated in a specific direction. Out of them, 10 sequences maintain speed-1 and the other set maintains speed-2. Thus, the training set is made up of 160 (20 x 8) sequences. The training is carried out for 1000 epochs. Before the testing phase, the training set is presented to the CPNW, and the winning cell plane for each translational direction is recorded and used as a label to estimate CPNW performance on the test set. Eight different cell planes showed maximum responses to 8 different translational motion directions provided in the input stimuli. Please note that CPNW is trained to recognize translational motion direction. We created a test set with 80 sequences (5 initial dot positions x 8 directions x 2 speeds) and presented it to the network.

As shown in Figs. S1A-H, each of the test sequences under the speed-1 category is responded to maximally and uniquely by one of the 8 cell planes. We see that though each cell plane responded most strongly to its preferred translational motion directions, it display different response profiles at different speeds. Figs. S1I-P shows CPNW responses to speed-2 translational motion sequences. CPNW showed 100% accuracy on the test set. Note that 100% accuracy is for testing the direction of motion and is independent of speed.

1.2. Three-stage network response/ *perceptron response to optic flow sequence*

Here we train the optic flow network (multi-class perceptron) simulating MST neurons and test the complete model-1 composed of all three stages: initial VSMN, middle CPNW, and output OFNW/ perceptron. The training set to train OFNW/ perceptron is composed of optic flow

sequences including contraction, expansion, clockwise rotation, and counter clockwise rotation each with 15 initial dot positions and 2 speeds. Thus, the training set is made up of 80 (10 dot positions x 4 flow types x 2 speeds) flow sequences. The OFNW/ perceptron is trained by repeatedly presenting sequences in the training set in a random order to the VSMN. While training the perceptron, the weights of VSMN and CPNW that were trained earlier were kept constant and only their responses were fed forwarded to OFNW/perceptron and trained for 500 epochs. Now the test set comprising 40 sequences (5 initial dots x 4 flow types x 2 speeds) was presented to model-1 and the responses are plotted in Fig. S2. Figs. S2A-D respectively shows the model-1 response to speed-1 anti-clockwise, clockwise, expansion (Zoom Out), and contraction (Zoom In) motions. Figs. S2E-H display model-1 response to speed-2 optic flow sequences. The accuracy obtained on the training set and test set is 100% and 85% respectively.

2. Model-2

2.1. Two-stage network response/ *HBNW response to translational motion*

The first stage in model-2 is replaced with VSMN. The second stage, HBNW, is made up of a 16 x 16 x 8 array of neurons. The training set consists of 160 translational sequences (10 positions x 8 directions x 2 speeds). HBNW is trained by repeatedly presenting translational motion sequences to VSMN, whose responses in turn were forwarded to HBNW neurons. Training is carried out for 10000 epochs (learning rate = 0.05). As a result of competitive learning, the neurons in the HBNW learn to encode the local flow direction together with the speed present in the small part of the image, and on the whole continuum of neurons (16 x 16 x 8) encodes global motion information present in the input sequence.

Trained HBNW responses to translational sequences of 180° and 315° are plotted in Fig. S3. Figs. S3A, D, G, and J display the last frame of a translational motion sequence, Figs. S3B, E, H, and K represent the corresponding VSMN response. To our surprise, VSMN produces different responses to different speeds. Figs. S3C, F, I and L displays HBNW responses. By comparing Figs. S3 C and F or I and L, one can observe that, at each (m, n) location along the z direction, only one neuron shows the highest response (winner) which varies for speed-1 and speed-2, implying HBNW encoding speed along with the direction. This might be due to variations in the VSMN input supplied during training.

Model-2 HBNW neurons described in the manuscript show the highest response when their preferred direction best fits the local motion direction in the input. On the other hand, here

HBNW neurons encode both direction and speed which resulted in a different set of winner neurons.

2.2. Three-stage network response/ *MLP response to optic flow motion*

Here we train the OFNW (multi-layer perceptron with the same architecture as described in the manuscript (Fig. 3)) simulating MST neurons and test model-2 composed of all the three stages: VSMN, HBNW and OFNW/ MLP. MLP is trained using a regular backpropagation algorithm for 5000 epochs (learning rate = 0.1). The activation function used by nodes in the hidden and output layers is sigmoid and SoftMax respectively. Note that the neuron responses produced by CPNW are very different from the responses produced in HBNW during competitive learning. As the nature of the input presented to the output stage varies in model-1 and 2, different classification algorithms were proposed for OFNW.

Training and test sets are composed of 80 and 40 flow sequences respectively, each including contraction, expansion, clockwise rotation, and anti-clockwise rotation as described in section 1.2. While training MLP, VSMN, and HBNW weights were kept constant. The accuracy obtained on the training set and test set is 100% and 82.5% respectively. Once MLP training is completed, the response of the three-stage network is observed for every sequence type in the test set and plotted in Fig S4.

Figure Captions

Fig. S1: **CPNW response to translational sequences:** In all figures from (A) to (P) a frame (80 x 80) of a translational sequence and its corresponding response on CPNW is plotted. The numbers 0,45,90 etc., represent the direction of motion of the input sequence. S1, S2 represents speed-1 and speed-2. CPNW consists of 8 cell planes, each showing maximum response to specific translational motion direction (irrespective of speed) as a result of training. The amount of activity produced by cell-plane neurons can be estimated using color bars. One can also observe that, though the same cell plane produces the highest activity in response to translational sequences having different speeds, their response profiles appear different.

Fig. S2: **Model-1 response to optic flow sequences:** trained three-layer network response to (A), (E) Anti-clock wise sequence (B), (F) clockwise sequence (C), (G) zooms out or radially outward sequence (D), (H) zoom in or radially inward sequence. In each case 'VSMN Resp' represent the populations of neurons that are active in each tile. In the 2nd and 4th columns 'CPNW Resp' represents the subset of neurons that are active in each Cell-plane in response to the given flow pattern when moved with speed-1 and speed-2 respectively. The responses on 8 cell planes are arranged as a 1D vector before giving it to OFNW. OFNW is an 8-class perceptron made up of 2 layers (input and output), and its response to a given flow sequence is

shown as 'OFNW Resp'. Here we displayed only the classification layer, where neurons 1-8 represent- speed1zoom in, speed1 Zoom out, speed1 clockwise rotation, speed1 anti-clockwise rotation, speed2zoom in, speed2 Zoom out, speed2 clockwise rotation, and speed2 anti-clockwise rotation respectively.

Fig. S3: **HBNW response to translational motion sequences:** Here we plotted HBNW response to four translational motion sequences: 180° and 315° (each with speed1 and speed2). (A), (D), (G), and (J) represent a frame in an input sequence, and the corresponding VSMN response is shown in (B), (E), (H), and (K). Hebbian network (16 x 16 x 8) response titled 'HBNW Resp' is plotted in (C), (F), (I), and (L). At each vertical column, the winner is highlighted with the arrow whose head indicates the neuron's direction preference. The color bar indicates the range of neuron activities produced by the given input.

Fig. S4: **Model-2 responses to optic flow sequences:** Trained three-layer network response to (A), (E) Anti-clock wise sequence (B), (F) clockwise sequence (C), (G) zoom out or radially outward sequence (D), (H) zoom in or radially inward sequence were shown. S1 and S2 indicate the optic flow sequences moved with speed-1 and speed-2 respectively. In each case 'VSMN Resp' represent the populations of neurons that are active in each tile to the input sequence. 'HBNW Resp' plots the Hebbian network response. The winner at each vertical column and its direction preference (arrow) were plotted. By comparing the 2nd and 4th column HBNW responses for each flow type, one can observe that through competitive learning different speed sequences were encoded by distinct clusters of HBNW neurons. 'OFNW Resp' represents the response of the output layer nodes in MLP, each node is encoding a specific flow type and of specific speed.
