# Supplementary figures and images for: Modeling the development of cortical responses in primate dorsal (“where”) pathway to optic flow using hierarchical neural field models
Source: Front Neurosci. 2023 May 22;17:1154252. doi: 10.3389/fnins.2023.1154252 (PMC10239834; doi:10.3389/fnins.2023.1154252)

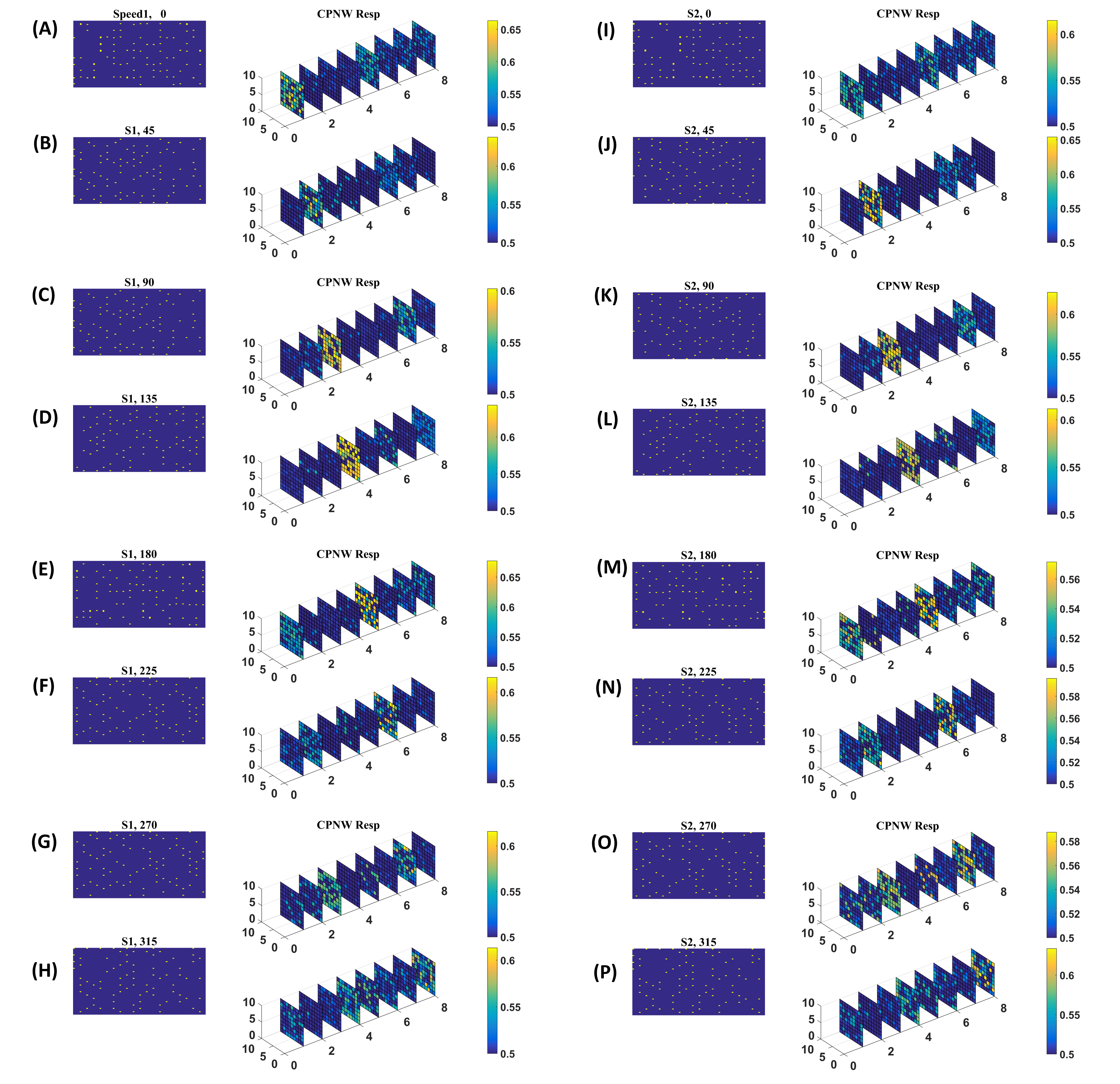

Supplement: Supplementary file 2 [file Image_1.TIF]

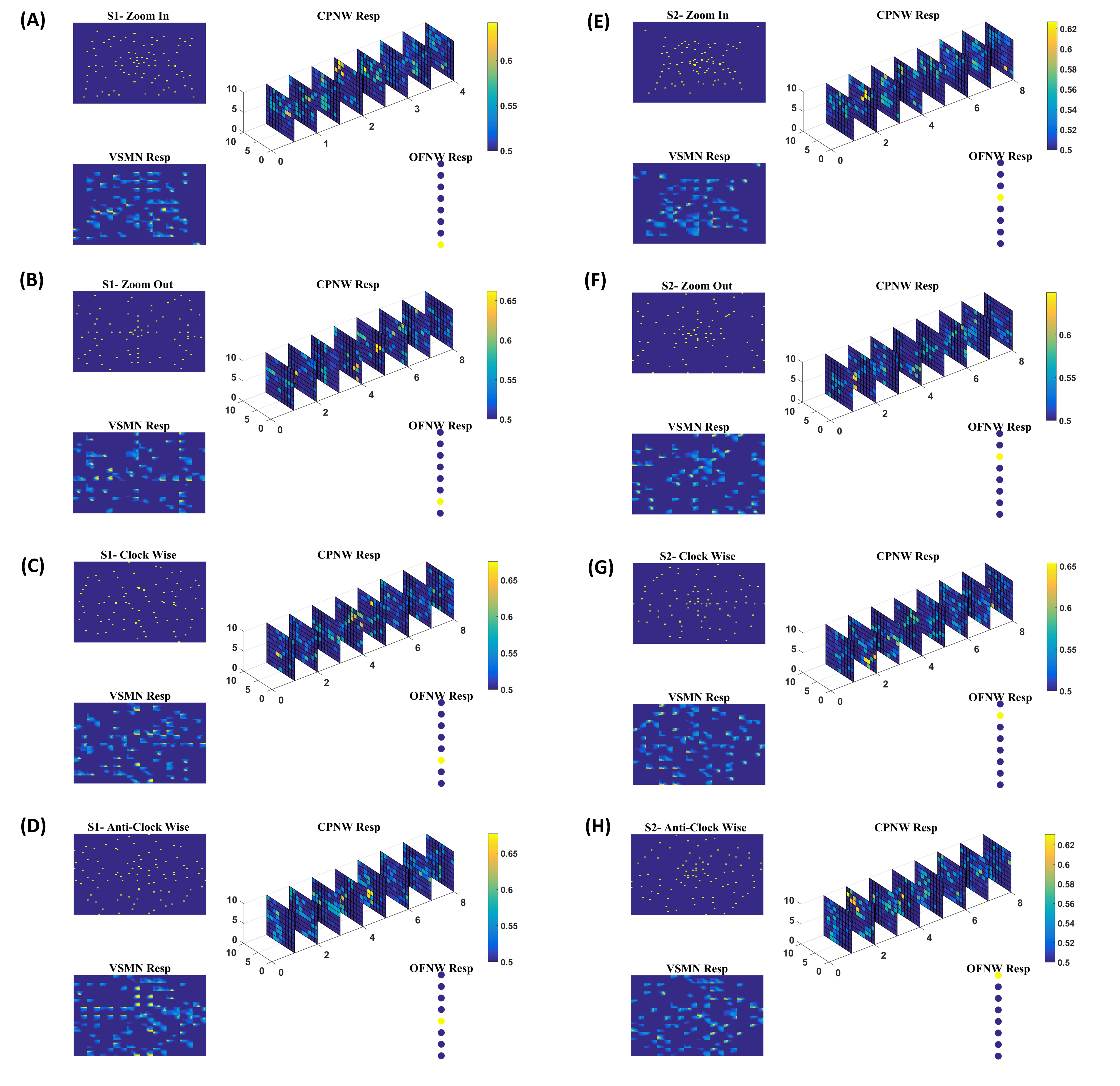

Supplement: Supplementary file 3 [file Image_2.TIF]

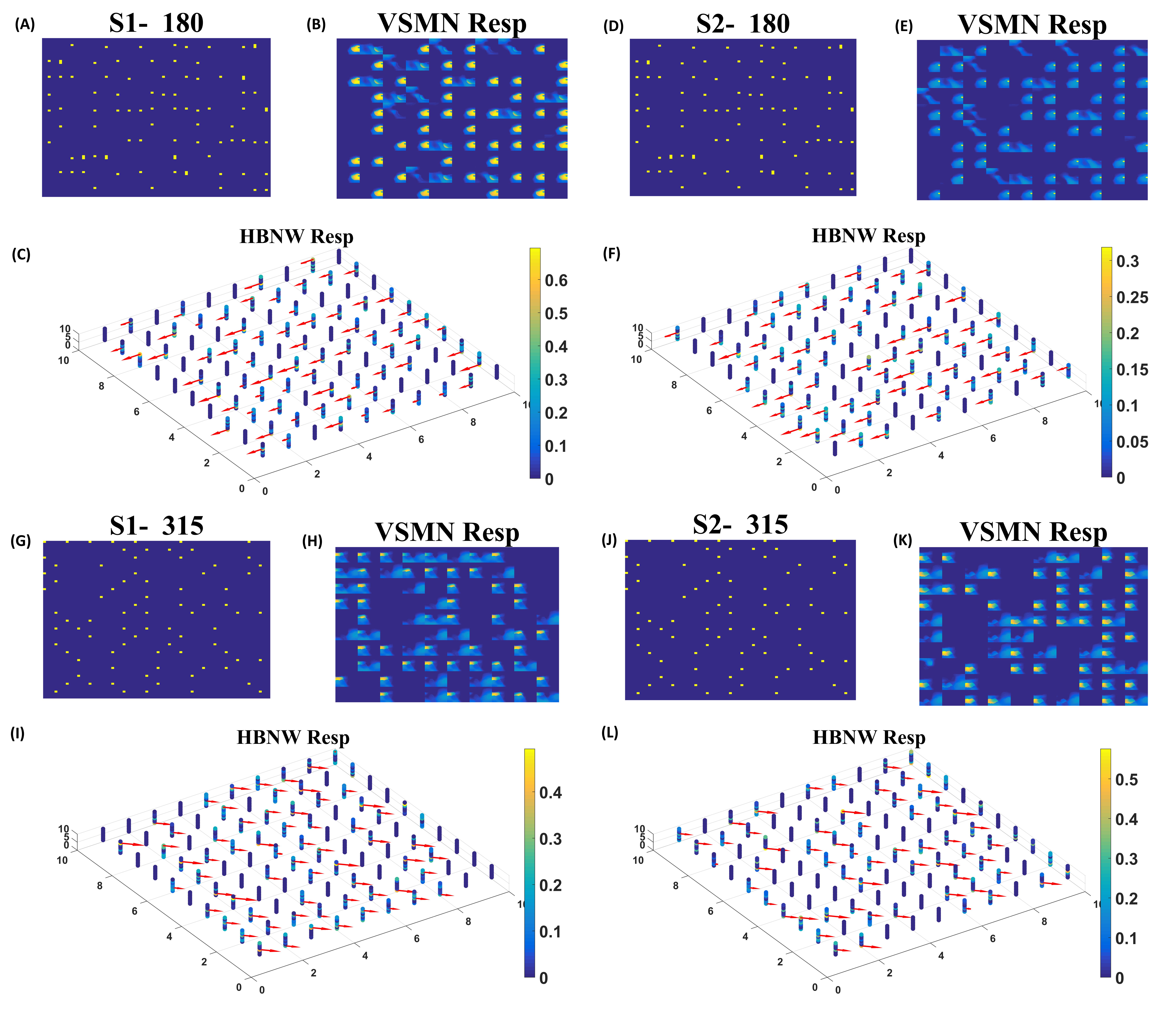

Supplement: Supplementary file 4 [file Image_3.TIF]

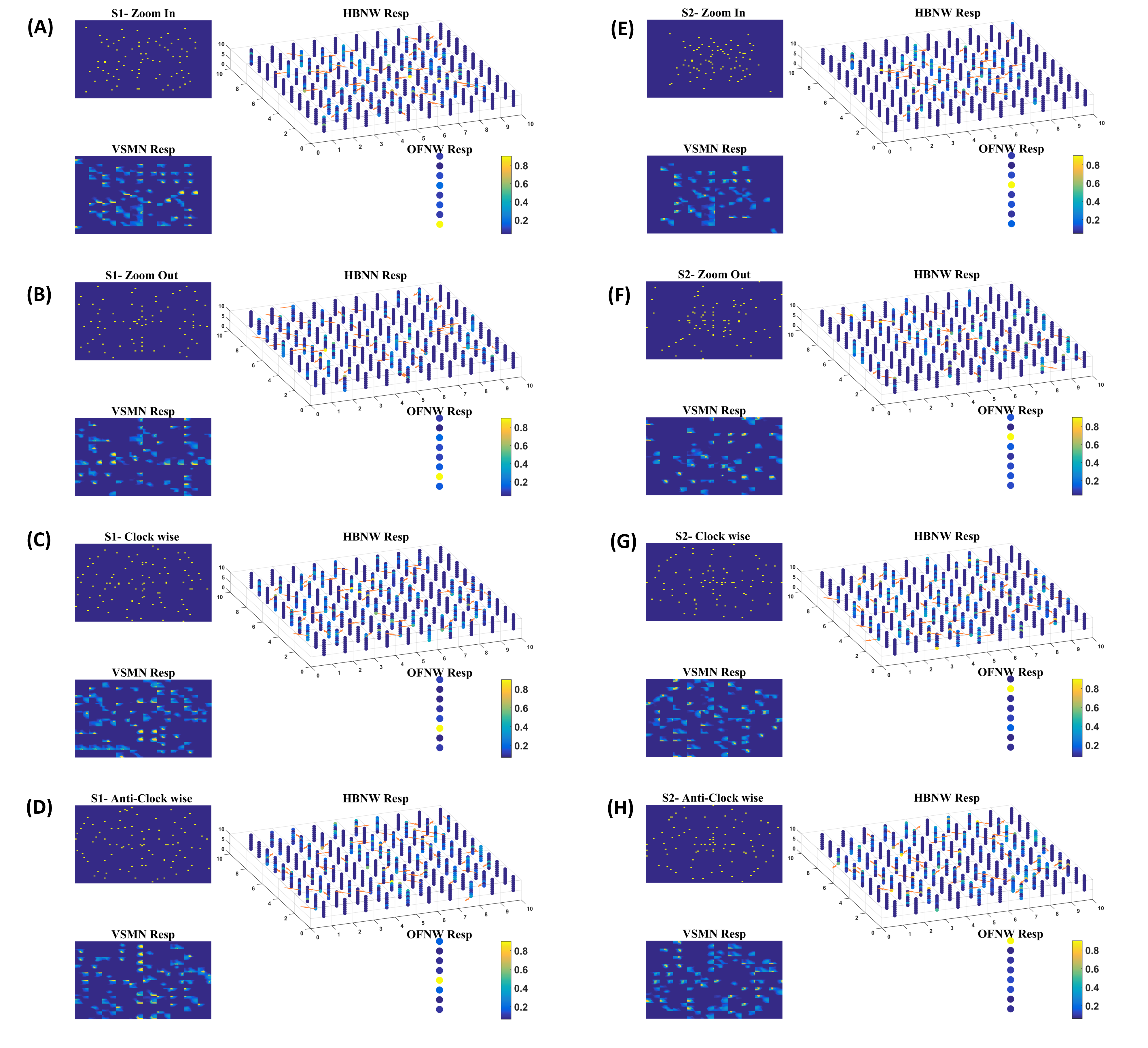

Supplement: Supplementary file 5 [file Image_4.TIF]
